# MCU expression in hippocampal CA2 neurons modulates dendritic mitochondrial morphology and synaptic plasticity

**DOI:** 10.1038/s41598-025-85958-4

**Published:** 2025-02-06

**Authors:** Katy E. Pannoni, Quentin S. Fischer, Renesa Tarannum, Mikel L. Cawley, Mayd M. Alsalman, Nicole Acosta, Chisom Ezigbo, Daniela V. Gil, Logan A. Campbell, Shannon Farris

**Affiliations:** 1 Fralin Biomedical Research Institute at Virginia Tech Carilion, Roanoke, VA USA; 2 Graduate Program in Translational Biology, Medicine, and Health, Virginia Tech, Blacksburg, VA USA; 3 Department of Biomedical Sciences and Pathobiology, Virginia-Maryland College of Veterinary Medicine, Virginia Tech, Blacksburg, VA USA; 4 Virginia Tech Carilion School of Medicine, Roanoke, VA USA

**Keywords:** Mitochondrial calcium uniporter, Hippocampal CA2, Synaptic plasticity, Mitochondria, Dendrites, Spines, Molecular neuroscience, Synaptic plasticity, Mitochondria

## Abstract

Neuronal mitochondria are diverse across cell types and subcellular compartments in order to meet unique energy demands. While mitochondria are essential for synaptic transmission and synaptic plasticity, the mechanisms regulating mitochondria to support normal synapse function are incompletely understood. The mitochondrial calcium uniporter (MCU) is proposed to couple neuronal activity to mitochondrial ATP production, which would allow neurons to rapidly adapt to changing energy demands. MCU is uniquely enriched in hippocampal CA2 distal dendrites compared to proximal dendrites, however, the functional significance of this layer-specific enrichment is not clear. Synapses onto CA2 distal dendrites readily express plasticity, unlike the plasticity-resistant synapses onto CA2 proximal dendrites, but the mechanisms underlying these different plasticity profiles are unknown. Using a CA2-specific MCU knockout (cKO) mouse, we found that MCU deletion impairs plasticity at distal dendrite synapses. However, mitochondria were more fragmented and spine head area was diminished throughout the dendritic layers of MCU cKO mice versus control mice. Fragmented mitochondria might have functional changes, such as altered ATP production, that could explain the structural and functional deficits at cKO synapses. Differences in MCU expression across cell types and circuits might be a general mechanism to tune mitochondrial function to meet distinct synaptic demands.

## Introduction

Mitochondria dynamically regulate many critical cellular functions, including energy production and calcium buffering, to meet the unique demands of different cell types^1–3^ . Even within a cell, mitochondria display remarkable heterogeneity across subcellular compartments^4–6^, which is especially critical for highly polarized cells such as neurons. The extent to which mitochondrial diversity influences cell-specific functions remains an open question. Mitochondrial dysfunction has been described in many neurological disorders, including Alzheimer’s disease^7–9^, schizophrenia^10–12^, autism spectrum disorder^13^, depression^14^ and bipolar disorder^15^. For this reason, it is critical to understand the relationship between mitochondria and neuronal function. In neurons, there is growing evidence demonstrating the importance of mitochondria for synapses and plasticity^16–20^. In particular, dendritic mitochondria in cultured hippocampal neurons are critical for maintaining spine density^17^ and for powering local-translation-dependent structural plasticity^21^. In acute slices, disrupting mitochondrial fission impairs long-term potentiation (LTP) at CA3-CA1 Schaffer collateral synapses^22^. However, the molecular mechanisms regulating the crosstalk between mitochondria and synapses are not well understood.

The mitochondrial calcium uniporter (MCU) is a channel that allows calcium flux across the inner mitochondrial membrane into the matrix of the mitochondria^23–25^, where it has potentially far-reaching effects on mitochondrial form and function^26–29^. While this has been extensively studied in pathological conditions and in culture^26,29–31^, little is known about the role mitochondrial calcium uptake plays in intact neural circuits. It’s thought that calcium influx via MCU may be a mechanism to couple neuronal activity to mitochondrial metabolism^32,33^, allowing neurons to rapidly adapt to changing energy demands. Previously, we found a striking enrichment of MCU in hippocampal area CA2^6,34^, a subregion of the hippocampus that is critical for social memory^35–37^. In contrast to CA1 neurons, CA2 neurons are known for being resistant to tetanus-induced LTP^38^, likely due to robust calcium buffering and extrusion mechanisms^39,53^ . While this is the case for CA3 synapses onto the proximal dendrites of CA2 (Schaffer collaterals, SC), entorhinal cortex layer II (ECII) synapses onto distal dendrites of CA2 (perforant path; PP) readily express LTP^35,40^. There are not any known candidates distinguishing CA2 proximal and distal synapses that could mediate these functional differences in plasticity. We recently uncovered that the propensity for LTP in CA2 correlates with more mitochondrial mass and higher expression of MCU selectively in distal dendrites compared to proximal dendrites^6^. The distinct enrichment of MCU in CA2 distal dendrites is not solely due to more mitochondrial mass, as other mitochondrial markers, such as COX4, do not show the same level of enrichment as MCU in CA2 distal dendrites and do not differ between areas CA1 and CA2^6^. Thus, we hypothesized that layer-specific enrichment of MCU in CA2 distal dendrites may be important for promoting plasticity in otherwise LTP-resistant CA2 neurons. Here, we generated a conditional knockout (cKO) of MCU in CA2 neurons and found MCU is necessary for LTP at ECII-CA2 distal synapses, but the lack of LTP at CA3-CA2 proximal synapses is unaffected by MCU loss. MCU deletion caused mitochondrial fragmentation across all CA2 dendritic layers, which did not alter the relative layer-specific mitochondrial structural diversity across CA2 dendrites. MCU loss also resulted in an overall decrease in average dendritic spine head area across CA2 dendrites in MCU cKO mice. These data expand on the underexplored role of MCU at the post-synapse in a cell type critical for social memory. Ultimately, understanding how diverse mitochondria regulate cellular functions to meet cell-type and circuit-specific needs is critical to our overall understanding of mitochondria in brain health and disease.

## Results

### Validation of conditional deletion of MCU in hippocampal CA2 neurons

**Fig. 1:**
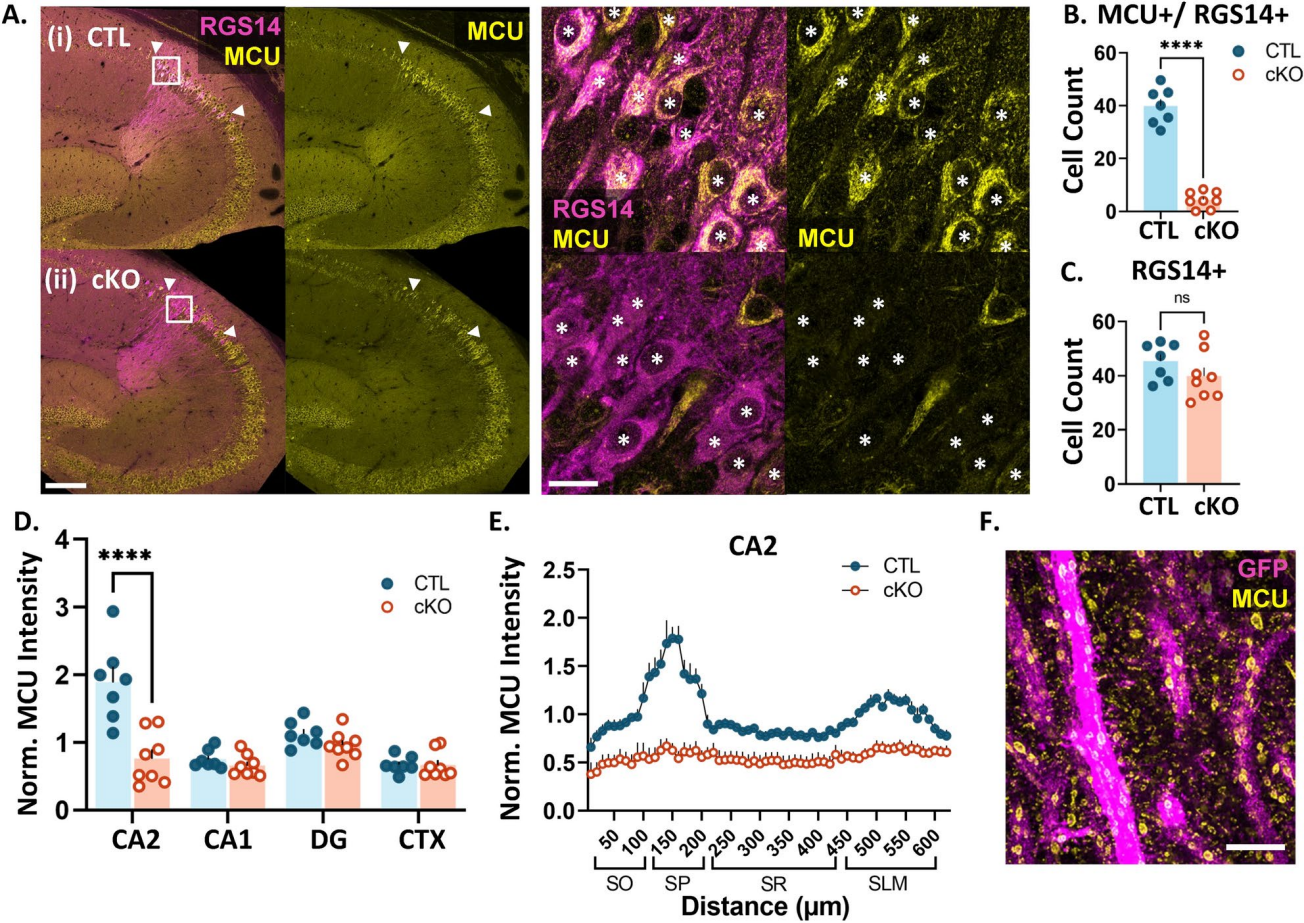
MCU expression is significantly reduced in CA2 neurons of cKO mice. (**A**) Representative images of RGS14 (magenta) and MCU (yellow) immunostaining in adult CTL (i) and cKO (ii) mice. Right: higher magnification images of CA2 neurons. Asterisks indicate RGS14-positive CA2 neurons. (**B**) Quantification of the number of RGS14 + CA2 neurons per section expressing MCU (CTL: 39.8 ± 2.6 neurons, N = 7 mice, cKO: 4.3 ± 1.1 neurons, N = 8 mice, two-tailed unpaired t-test, *p* < 0.0001). (**C**) The total number of RGS14-positive CA2 neurons per section does not differ between CTL and cKO mice (CTL: 45.4 ± 2.6 neurons, cKO: 39.9 ± 3.1, two-tailed unpaired t-test, *p* = 0.204). (**D**) MCU fluorescence intensity in CTL and cKO mice in CA2, CA1, dentate gyrus (DG) and neighboring cortex (CTX). Data were normalized to the CTL average. (two-way ANOVA, overall effect of genotype F (1, 52) = 22.45, *p* < 0.0001; overall effect of subregion F (3, 52) = 17.80, *p* < 0.0001, interaction F (3, 52) = 12.74, *p* < 0.0001; sidak’s post hoc test CTL v. cKO CA2 *p* < 0.0001). (**E**) Line plot analysis of MCU fluorescence intensity across CA2 layers in same CTL and cKO mice. Data are normalized to the CA2 CTL average. (**F**) Representative super resolution image of MCU (yellow) in genetically labeled CA2 dendrites (magenta) using expansion microscopy (ExM). Scale = 200 µm and 25 µm (**A**) and 5 µm, ExM adjusted (**F**). ****p < 0.0001 Data points are individual animal averages ± sem, except in (**E**) where the data points are averaged across animals ± sem.

To examine whether MCU plays a role in the layer-specific plasticity profile of CA2, we generated a CA2-specific cKO mouse of MCU by crossing an Amigo2-cre mouse line^41^ to a floxed MCU line (MCU^fl/fl^,^42^). Sections from adult MCU^fl/fl^;Amigo2-cre negative (CTL) and MCU^fl/fl^;Amigo2-cre positive (cKO) mouse brains were immunostained for MCU and CA2 neuronal marker RGS14 to validate the selective loss of MCU in CA2 neurons. On average, 88% (± 2.2, N = 7 mice) of RGS14 positive CA2 neurons express MCU in CTL mice. After postnatal MCU deletion, 10% (± 2.7, N = 8 mice) of RGS14 positive CA2 neurons express MCU (Fig. 1A,B). Consistently, there was a significant decrease in the number of RGS14 positive neurons per section that express MCU in cKO mice compared to CTL mice (Fig. 1B; CTL: 39.8 ± 2.6 neurons, cKO: 4.3 ± 1.1 neurons, two-tailed unpaired t-test, *p* < 0.0001) without a difference in the total number of RGS14 positive CA2 neurons per section between genotypes (Fig. 1C; CTL: 45.4 ± 2.6 neurons, cKO: 39.9 ± 3.1, two-tailed unpaired t-test, *p* = 0.204) MCU fluorescence intensity was reduced selectively in CA2 neuron cell bodies (Fig. 1D; two-way ANOVA, overall effect of genotype F (1, 52) = 22.45, *p* < 0.0001, sidak’s post hoc test CTL v. cKO CA2 *p* < 0.0001), with no significant change in MCU fluorescence intensity in cell bodies in CA1, dentate gyrus (DG), or the neighboring cortex in MCU cKO compared to CTL (sidak’s post hoc CTL v. cKO CA1, DG, CTX *p* > 0.05). Further, a decrease in MCU fluorescence intensity was seen across all layers of CA2 dendrites in the cKO compared to CTL (Fig. 1E). In the neuropil layer, MCU labeling is predominantly detected within dendrites, where it localizes to the inner mitochondrial membrane as visualized with protein-retention expansion microscopy (Fig. 1F). We previously showed that MCU colocalizes with a mitochondrial matrix marker in expanded confocal images^6^, verifying that the staining is localized to the inner mitochondrial membrane.

**Fig. 2:**
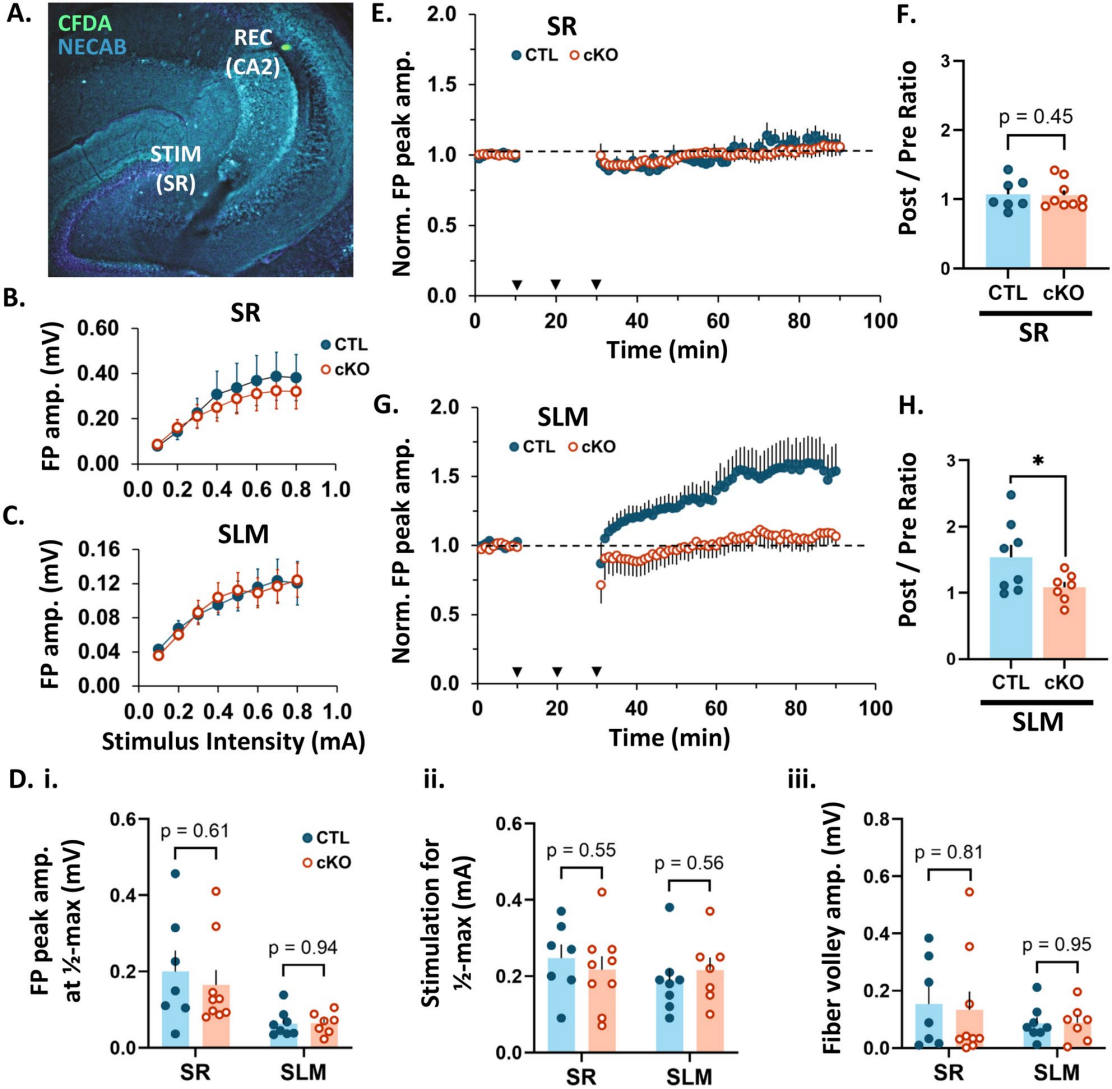
CA2 MCU cKO blocks LTP in CA2 SLM, with no effect in plasticity-resistant CA2 SR. (**A**) Representative image of the recording and stimulating site in an acute hippocampal slice. CFDA dye (green) was pressure ejected from the recording pipette. NECAB staining (cyan) delineates CA2. (**B**) Input–output curve showing the relationship between field potential amplitude and stimulus intensity after stimulation of CA3 inputs to SR of CTL (blue, closed) and cKO slices (orange, open). No significant difference in response amplitude at any stimulus intensity between CTL and CKO slices (*p* > 0.05, Welch’s two-tailed t-tests; N = 7 CTL, 9 cKO slices). (**C**) Input-output curve after stimulation of ECII inputs to SLM of CTL and cKO slices. No significant difference in response amplitude at any stimulus intensity between CTL and CKO slices (*p* > 0.05, Welch’s two-tailed t-tests; N = 8 CTL, 7 cKO slices). (**D**) (i) Barplot showing peak field potential amplitude at 1/2 max stimulus intensity in CTL and cKO slices. All statistics in D are two-tailed Welch’s t-tests (N = SR: 7 CTL, 9 cKO; SLM: 8 CTL, 7 cKO slices). (ii) Barplot showing the stimulation required to produce a 1/2 max response in CTL and cKO slices. (iii) Barplot showing peak fiber volley amplitude at 1/2 max stimulus intensity in CTL and cKO slices. (**E**) Average time plot of normalized field potential peak amplitudes evoked in CA2 by stimulation of CA3 inputs to SR of CTL and MCU cKO slices. N = 7 CTL, 9 cKO slices. Dotted black line = baseline (all plots). Error bars = SEM (all plots). Arrow heads = stimulation at 100 Hz for 1 s; 3 bursts with 10 min interval. (**F**) Post/pre ratio of normalized field potential peak amplitudes evoked in CA2 by stimulation of CA3 inputs to SR of CTL and cKO slices. One-tailed Welch’s t-test; N = 7 CTL, 9 cKO slices. (**G**) Average time plot of normalized field potential peak amplitudes evoked in CA2 by stimulation of ECII inputs to SLM of CTL and MCU cKO slices. N = 8 CTL, 7 cKO slices. (**H**) Post/pre ratio of normalized field potential peak amplitudes evoked in CA2 by stimulation of ECII inputs to SLM of CTL and cKO slices. One-tailed Welch’s t-test; N = 8 CTL, 7 cKO slices. *p < 0.05.

### CA2-specific MCU cKO results in impaired LTP at distal dendrite synapses

Next, to assess the role of MCU in the propensity of CA2 distal synapses to express LTP, we recorded extracellular field potentials (FP) from CA2 neurons in acute hippocampal slices from adult CTL and cKO mice. A stimulating electrode was placed in either the Schaffer collateral (SC) inputs from CA3 to CA2 stratum radiatum (SR, proximal dendrites) or the perforant path (PP) inputs from ECII to CA2 stratum lacunosum moleculare (SLM, distal dendrites) and the recording electrode was placed in the CA2 stratum pyramidal (Fig. 2A). The recording site was marked by ejection of CFDA (a green fluorescent dye) for confirmation of placement within CA2. First, we examined whether baseline synaptic responses were altered by MCU cKO by comparing stimulus- response relationships (input-output curves) for evoked FP peak amplitudes recorded in slices from CTL and cKO mice. No significant differences were found between CTL and cKO slices in the overall input–output curves for stimulation of either CA3 inputs to SR (*p* > 0.05 for all stimulus intensities, two-tailed Welch’s *t*-tests, Fig. 2B) or ECII inputs to SLM (*p* > 0.05 for all stimulus intensities, two-tailed Welch’s *t*-tests; Fig. 2C). Further, during the 10 min pre-conditioning baseline, we found no significant difference between CTL and cKO slices in: the half-maximal evoked FP peak amplitude (Fig. 2D (i); CA3 inputs to SR: *p* = 0.61; ECII inputs to SLM: *p* = 0.94; two-tailed Welch’s *t*-tests), the stimulation intensity required to produce half-maximal responses (Fig. 2D (ii); CA3 inputs to SR: *p* = 0.55; ECII inputs to SLM: *p* = 0.56; two-tailed Welch’s *t*-tests), or in the peak fiber volley amplitude produced by half-maximal stimulation (Fig. 2D (iii); CA3 inputs to SR: *p* = 0.81; ECII inputs to SLM: *p* = 0.95; Welch’s *t*-tests). These results show that basal synaptic transmission is not altered by MCU deletion in CA2.

For LTP induction experiments, a prerequisite stable 10-min pre-conditioning baseline was obtained at 0.1 Hz. This was followed by delivery of a strong tetanizing stimulation consisting of three bursts of high frequency stimulation (3 × 100 Hz for 1 s) with an inter-burst interval of 10 min. Subsequently, post-conditioning responses were recorded for a period of 60 min at 0.1 Hz. Only one recording was made from each slice. Figure 2D shows the average normalized field potential peak amplitude evoked by stimulation of CA3 inputs to SR over time in the CTL and cKO conditions. The ratio of the normalized field potential peak amplitude during the last 5 min of post-conditioning/last 5 min of pre-conditioning (post/pre ratio) is also plotted in Fig. 2E. Consistent with observations in wildtype mice^38^, stimulation of CA3 inputs to the SR of CTL mice did not induce a net LTP and this did not change in cKO mice (Fig. 2E,F; average post/pre ratio: CTL = 1.07 + / − 0.08, n = 7 slices from 7 mice, cKO = 1.06 + / − 0.07, n = 9 slices from 9 mice; *p* = 0.45, one-tailed Welch’s *t*-test). In contrast to CTL mice, where stimulation of ECII inputs to SLM induced robust LTP as previously described ^40^, stimulation of ECII inputs to SLM failed to induce a robust LTP in cKO mice (Fig. 2G,H, average post/pre ratio: CTL = 1.54 + / − 0.19, n = 8 slices from 7 mice, cKO = 1.08 + / − 0.08 n = 7 slices from 7 mice; p = 0.03, one-tailed Welch’s *t-*test). Individual time plots of normalized FP peak amplitude over time are presented for each slice in Supplemental Fig. 1. We saw a heterogeneous response to strong tetanizing stimulation with a variety of post/pre ratios (plasticity outcomes). For stimulation of CA3 inputs to SR in CTL and cKO slices, the majority of responses showed no change (post/pre ratios within + / − 10% of baseline), while a single slice in each group exhibited long-term depression (LTD, post/pre ratio < 90% of baseline), and 33–43% displayed weak LTP (post/pre ratio = 114–143% of baseline). This heterogeneity resulted in no net change from baseline, which is consistent with previous reports^38^. In contrast, after stimulation of ECII inputs to SLM in CTL slices, 75% of recordings showed robust LTP (post/pre ratios = 120–248% of baseline) and none showed LTD, while in cKO slices 57% displayed weak LTP (post/pre ratios = 112–138% of baseline) and 14% displayed LTD. Combined, these results confirm layer-specific plasticity profiles in CTL CA2 and demonstrate that MCU deletion impairs the capacity of synapses in SLM to undergo robust LTP.

### CA2 MCU cKO alters dendritic mitochondrial morphology and content

**Fig. 3:**
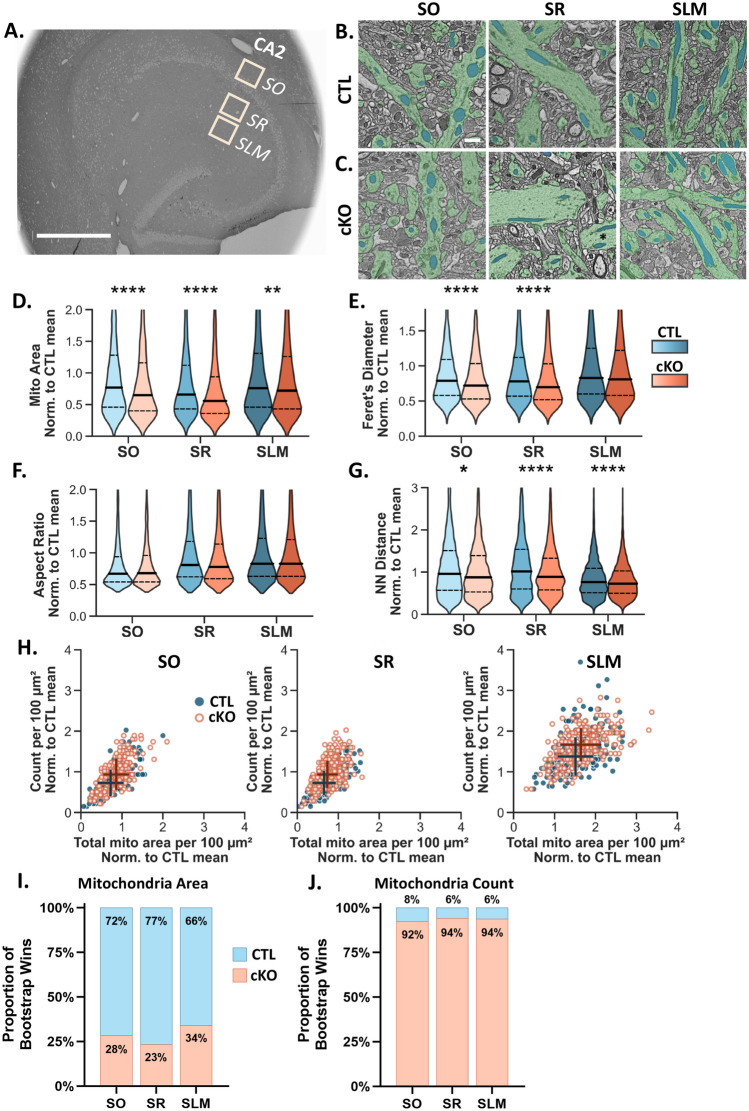
Dendritic mitochondria in MCU cKO mice are smaller and more numerous than CTL mice. (**A**) Representative scanning electron microscopy (SEM) overview of a horizontal hippocampal section. Boxes represent the sampling areas from CA2 SO, SR and SLM. Scale = 600 µm for all images. (**B**) Representative SEM images from CA2 SO, SR, and SLM of CTL showing dendritic mitochondria (blue) and dendrites (green). Scale = 1 µm for (**B**–**C**). (**C**). Representative SEM images from CA2 SO, SR and SLM of the MCU cKO. Black asterisk shows an example of mitochondria in a stacked orientation. (**D**) Violin plots of individual mitochondrial area in 100 μm^2^ image tiles from CA2 SO, SR and SLM in CTL (blue) and cKO mice (orange). All plots were normalized to the overall mean of the CTL. CTL SO n = 2353, CTL SR n = 2904, CTL SLM n = 5236. cKO SO n = 2860, cKO SR n = 4007, cKO SLM n = 6299 mitochondria. Solid line = median; dashed line = upper and lower quartiles. All medians and standard deviations are listed in Table 1 for this figure. (**E**). Normalized mitochondria Feret’s diameter from the same dataset in (**D**). (**F**) Normalized mitochondria aspect ratio from the same dataset in (**D**). See Supplemental Fig. 2 for pairwise layer comparisons in the CTL. (**G**) Normalized mitochondria nearest neighbor distance from the same dataset in (**D**). (**H**) Correlation plot comparing normalized mitochondrial count and normalized total mitochondrial area per 100 μm^2^ in CA2 SO, SR, and SLM in CTL (blue, closed) and MCU cKO (orange, open). CTL SO n = 223, CTL SR n = 279, CTL SLM n = 260. cKO SO n = 218, cKO SR n = 298, cKO SLM n = 279, n = 100 μm^2^ tiles. + indicates the median of each group with horizontal and vertical lines indicating standard deviation for each axis. (**I**) The proportion of bootstrap iterations (n = 10,000) where median mitochondria area was greater in the CTL (blue) or the cKO (orange) for each layer of CA2. (**J**) The proportion of bootstrap iterations (n = 10,000) where the median number of mitochondria per tile was greater in the CTL (blue) or the cKO (orange) for each layer of CA2.

Studies have shown the importance of mitochondria both pre- and post-synaptically to support synaptic function and plasticity^17–19,21^. We previously showed that the propensity for LTP at CA2 distal dendrite synapses corresponds with a layer-specific enrichment of MCU and larger mitochondria relative to the other dendritic layers^6^, which in theory could produce more ATP^5^. Calcium influx into mitochondria regulates both mitochondrial bioenergetics and the balance of fission/fusion^26,29^, thus we hypothesized that MCU deletion may lead to changes in mitochondrial ultrastructure that could explain a plasticity deficit in CA2. To look at changes in mitochondrial morphology after MCU deletion, we compared mitochondrial ultrastructure in scanning electron microscopy images from CTL and cKO mice. We acquired images from large (150 × 150 μm^2^) regions of interest (ROIs) from CA2 stratum oriens (SO; basal dendrites), SR (proximal dendrites) and SLM (distal dendrites) of each genotype (Fig. 3A). A deep learning Artificial Intelligence (AI) platform was used to selectively segment dendritic mitochondria in a subset of image tiles from each ROI totaling 155,200 µm^2^ in area. From a spot check, the AI correctly identified an estimated 92.2% of dendritic mitochondria, with an error rate of 2 errors/100 µm^2^ area.

In CTL mice, we quantified the previously characterized mitochondrial structural heterogeneity across CA2 dendritic layers (Supplemental Fig. 2A). Mitochondria in SO of CTL mice were uniquely rounded relative to SR and SLM, with an aspect ratio closer to 1 (Supplemental Fig. 2B and Table 1). We confirmed our previous findings based on manual segmentation that mitochondria in SLM of CTL CA2 are larger in area than mitochondria in SR (Supplemental Fig. 2C and Table 1)^6^. In CTL CA2 SLM, the median distance between neighboring mitochondria was shorter than mitochondria in SO or SR (Supplemental Fig. 2D and Table 1). This may be related to the fact that mitochondria were also longer in CA2 SLM compared to mitochondria in SO and SR (Supplemental Fig. 2E and Table 1) and harbor a greater overall mitochondrial content, as measured by mitochondrial count per 100 μm^2^ and total mitochondrial area per 100 μm^2^ (Supplemental Fig. 2F and Table 1). We note that the relative increase in Feret’s diameter in SLM is greater when looking at the mean Feret’s diameter across layers (SO: 0.68 µm; SR: 0.72 µm; SLM: 0.81 µm) instead of the medians (SO: 0.59 µm; SR: 0.58 µm; SLM: 0.62 µm). A shift towards higher Feret’s diameter in SLM was seen for all quartiles. Thus, mitochondria in the proximal (SR) and distal (SLM) dendrites are distinguished by their differences in mass, diameter and overall content (Supplemental Fig. 2F), whereas mitochondria in the basal dendrites (SO) are distinguished mostly by their aspect ratio (Supplemental Fig. 2B).

**Table 1:** Summary table for the SEM dataset showing the medians, standard deviation, and N of each metric of interest from the analysis of mitochondria and dendritic spines. This is the summarized data plotted in Figs. 3 and 4.

	CTL			cKO		
Median (std)	SO	SR	SLM	SO	SR	SLM
N Mice	3	3	3	3	3	3
N Mitochondria	2353	2904	5236	2860	4007	6299
Mitochondria area (µm^2^)	0.155 (0.16)	0.134 (0.15)	0.153 (0.19)	0.131 (0.15)	0.112 (0.14)	0.145 (0.19)
Feret’s diameter (µm)	0.59 (0.36)	0.58 (0.48)	0.62 (0.56)	0.54 (0.37)	0.52 (0.45)	0.61 (0.56)
Aspect ratio	1.6 (1.09)	1.9 (1.6)	1.9 (1.63)	1.6 (1.17)	1.8 (1.55)	1.9 (1.66)
NN distance (µm)	1.47 (1.15)	1.57 (1.15)	1.19 (0.69)	1.36 (1.06)	1.37 (0.95)	1.13 (0.65)
Mito count per 100 µm^2^	10 (4.4)	10 (4.0)	19 (6.5)	13 (5.3)	13 (4.6)	23 (5.6)
Total mito area per 100 µm^2^	2.0 (0.9)	1.8 (0.8)	4.2 (1.2)	2.4 (0.9)	2.0 (0.7)	4.6 (1.4)
N image tiles	221	277	260	218	297	279
N′ mice	2	2	2	3	3	3
N′ spine heads	1141	1461	1842	1513	2412	2753
Spine head area (µm^2^)	0.146 (0.07)	0.138 (0.07)	0.145 (0.07)	0.131 (0.06)	0.126 (0.06)	0.134 (0.06)
Spine count per 100 µm^2^	11 (4.0)	12 (3.9)	15 (5.8)	11 (4.6)	12 (5.2)	15 (5.2)
N′ image tiles	103	124	121	145	197	185

N′ = subset used for dendritic spine analysis.

Due to the hierarchical nature of this dataset, assuming that individual mitochondria are statistically independent would result in false positives. To address this, we applied Bayesian statistics with a hierarchical bootstrap^43^ (Supplemental Fig. 3). The data were randomly resampled (with replacement) at the level of animal, hippocampal section, image tile, and individual mitochondria, and the median of the resampled data was calculated for each layer in CA2. This was repeated 10,000 times to get a population of resampled medians for each group, which were plotted together on joint probability distribution plots for each comparison. Consistent with our previous manual quantification^6^, the median mitochondria area was greater in CA2 SLM of CTL compared to SR (Supplemental Fig. 3A(iii–iv); SLM area was larger in 83% of iterations). In addition, mitochondria count per tile was greater in SLM compared to SR and SO in 100% of bootstrap iterations (Supplemental Fig. 3B). Our data from CTL mice confirm that the AI detects the established morphological diversity of mitochondria across the dendritic layers of CA2.

To determine whether loss of MCU affects mitochondrial ultrastructure, we compared dendritic mitochondria morphology in each layer of CA2 in CTL and MCU cKO mice (Fig. 3). In MCU cKO mice, individual mitochondria were on average smaller (Fig. 3D and Table 1) and shorter in the long axis (Feret’s diameter, Fig. 3E and Table 1) across all dendritic layers compared to CTL mice, with no changes in aspect ratio (Fig. 3F and Table 1). This indicates that mitochondria are smaller in both dimensions in cKO relative to CTL. There was also an increase in median mitochondrial count per 100 μm^2^ in all dendritic layers of cKO mice (Fig. 3H and Table 1). A decrease in individual mitochondria area in combination with an increase in mitochondria count suggests that MCU cKO causes an increase in mitochondria fragmentation. The increase in mitochondrial count, while greatest in SLM, appears to be driving an increase in overall dendritic mitochondrial content after MCU cKO (Fig. 3H). Strikingly, however, the relative differences in mitochondria size and mitochondrial content between dendritic layers were maintained in cKO as in CTL mice (CTL Supplemental Fig. 3A,B(iv) and cKO Supplemental Fig. 4A,B(iv)). These data suggest that MCU is not essential for the localization of larger, more numerous mitochondria to distal dendrites.

**Fig. 4:**
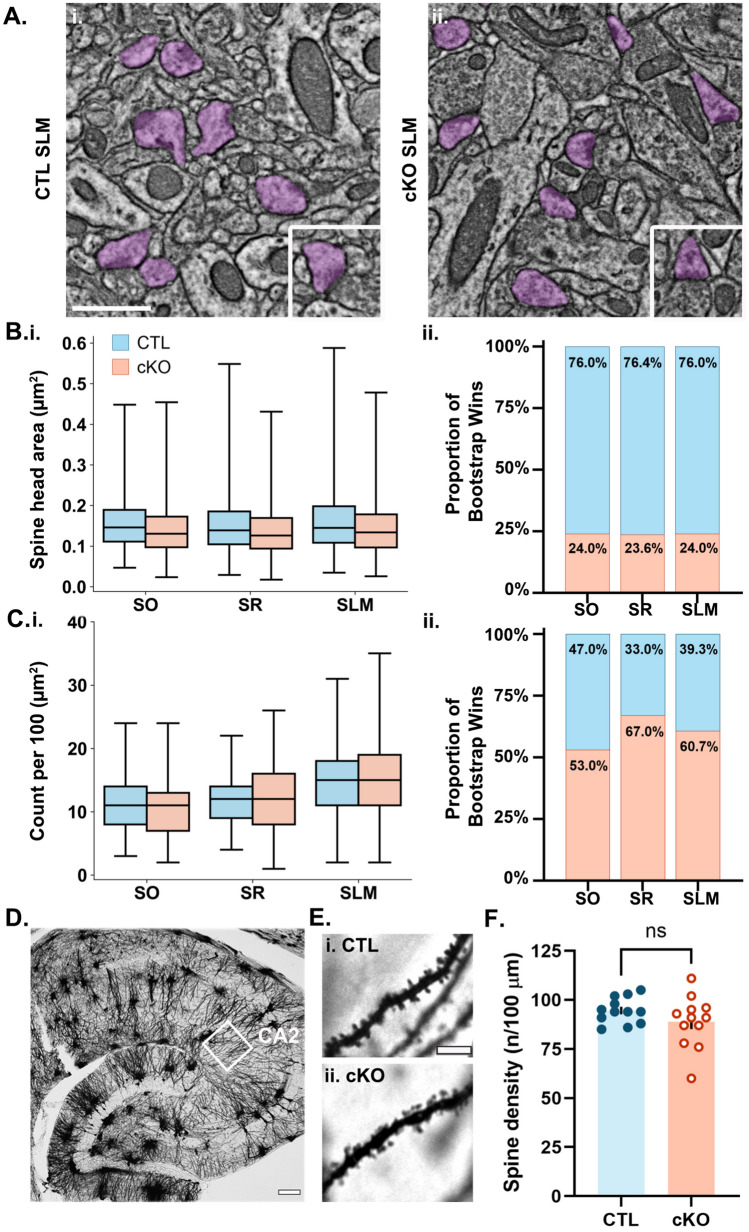
MCU deletion in CA2 decreases dendritic spine head size but does not alter spine density. (**A**) Representative SEM images from CA2 SLM of (i) CTL and (ii) cKO mice. Dendritic spine heads are highlighted in purple. Inset shows an example spine from another area of the same image tile. Scale = 1 μm; Inset = 1 μm x 1 μm. (**B**) (i) Boxplots of individual spine head area in CA2 SO, SR and SLM of the CTL (blue) and cKO mice (orange). CTL SO n = 1141, CTL SR n = 1461, CTL SLM n = 1842. cKO SO n = 1513, cKO SR n = 2412, cKO SLM n = 2753 dendritic spines from 2 CTL and 3 cKO mice. Center line = median. See Table 1 for medians and standard deviations. (ii) The proportion of bootstrap iterations (n = 10,000) where median spine head area was greater in the CTL (blue) or the cKO (orange) for each layer of CA2. (**C**) (i) Boxplots of spine head count per 100 μm^2^ in CA2 SO, SR and SLM of the same CTL and cKO mice. CTL SO n = 103, CTL SR n = 124, CTL SLM n = 121. cKO SO n = 145, cKO SR n = 197, cKO SLM n = 185 image tiles. See Table 1 for medians and standard deviations. (ii) The proportion of bootstrap iterations (n = 10,000) where median spine head count was greater in the CTL (blue) or the cKO (orange) for each layer of CA2. (**D**) 63X magnification image of Golgi-Cox stained mouse hippocampus. White box indicates CA2 SLM dendrites. Scale = 100 μm. (**E**) Representative Golgi-Cox images of (i) CTL and (ii) cKO SLM dendrites. Scale = 5 μm. (**F**) Quantification of average spine density per 100 μm dendrite in SLM of CTL and cKO. Each data point is an average of 12 dendrites from each mouse. There was no significant difference between genotypes (*p* = 0.199; Welch’s t-test; 12 mice per group). However, there was a significant increase in variability across animals in MCU cKO mice compared to CTL (*p* = 0.028, F = 4.056.77; F test for variance).

Paradoxically, there was also a decrease in median nearest neighbor distance in all dendritic layers of cKO mice compared to CTL mice (Fig. 3G and Table 1). We qualitatively observed more examples of cKO mitochondria that formed a stacked distribution within dendrites, resembling multiple lanes of traffic, compared to CTL mitochondria that typically formed a more linear network, oriented end to end (see Fig. 3BC SR and SLM). This altered network distribution could conceivably contribute to the decrease in the nearest neighbor distance in cKO compared to CTL (Fig. 3G). It is not yet clear the functional significance, if any, of this change.

Based on the observed increase in mitochondrial fragmentation, we also performed a hierarchical bootstrap to compare mitochondria area and count per tile in CTL and cKO mice (Fig. 3I,J; Supplemental Fig. 4). Joint probability distributions of the bootstrap medians were plotted comparing across layers within the cKO (Supplemental Fig. 4A–B) and to each CTL layer (Supplemental Fig. 4C–D). The resampled median mitochondria area was smaller in the cKO than the CTL in SO in 72% of the bootstrap iterations, 77% for SR, and 66% for SLM (Fig. 3I; Supplemental Fig. 4C), indicating that the probability that mitochondria are uniformly smaller in the cKO compared to CTL is greater than chance. The resampled median number of mitochondria per tile was greater in the cKO than the CTL in SO in 92% of the bootstrap iterations, 94% for SR, and 94% for SLM (Fig. 3J; Supplemental. Figure 4D), indicating that there is a very high probability that mitochondria are consistently more numerous in the cKO compared to CTL.

Taken together, our data suggest that mitochondrial fragmentation is increased in MCU cKO in CA2 compared to CTL, and this effect was seen across all dendritic layers. Although we saw no obvious signs of damaged or unhealthy mitochondria in the MCU cKO condition at the ultrastructural level, there may be functional changes or deficits that would not be observed in EM micrographs, such as decreased metabolism and ATP production.

### CA2 MCU cKO reduces spine head area but not density in distal dendrites.

LTP involves the strengthening of existing synapses as well as growth and stabilization of new spines and synapses^44–46^, thus we hypothesized there may be an alteration to spines associated with the LTP deficit and mitochondrial fragmentation in CA2 SLM. In a subset of our SEM dataset with sufficient resolution (N = 2 CTL, 3 cKO mice), a separate AI was trained to segment dendritic spine heads in SO, SR and SLM of CA2 in CTL and cKO (Fig. 4A), and spine head area and number were quantified across dendritic layers and genotypes. Spine head area has been shown to correlate with postsynaptic density (PSD) size and synapse strength^47^, and a decrease in synapse strength could cause a deficit in LTP. We found that median spine head area was smaller in cKO SLM compared to CTL SLM (Fig. 4B, Table 1). We applied a similar Bayesian statistical bootstrap as described above on individual spine head area, which revealed a 76% probability that spine head size is smaller in the cKO compared to CTL across all dendritic layers (Fig. 4B(ii) and Supplemental Fig. 5A–C), indicating that MCU deletion causes an overall decrease in the average dendritic spine head size.

To determine the effect of MCU deletion on the density of dendritic spines, we quantified the number of spine heads per 100 µm^2^ in the same dataset. Given that the cKO was over-represented in our subsetted SEM dataset, dendritic spine head density was normalized by area. In CTL mice, the density of spine heads was highest in SLM (Fig. 4C, Supplemental Fig. 5D, Table 1). We applied a similar Bayesian statistical bootstrap as described above on spine head density (Fig. 4C(ii) and Supplemental. Figure 5D-E), and found the density of spine heads was greater in SLM compared to SO or SR (SLM greater than SO: 98% of iterations; SLM greater than SR: 94% of iterations), indicating a very high probability that CTL CA2 SLM has more dendritic spines than SO or SR. This is consistent with previous studies showing that input onto the distal dendrites drives CA2 pyramidal neurons more strongly than the input onto their proximal dendrites^40^. We then compared spine head density in the cKO and CTL and did not find a difference in spine density across layers (Fig. 4C). The median spine head areas were nearly identical across genotypes (Table 1) and the probability that spine head density differed between the CTL and cKO was near chance for each layer (Fig. 4C(ii), % iterations greater in CTL, SO: 47%; SR: 33%; SLM: 39%).

To confirm that spine density is not altered in the cKO, we impregnated hippocampal sections from CTL and MCU cKO mice with Golgi-Cox staining solution^48^, a mercury-based sparse cell fill, and quantified the density of dendritic spines in CA2 SLM. Figure 4D–E shows a Golgi-Cox stained hippocampal section highlighting CA2 and representative dendrite segments from CA2 SLM of CTL and cKO. We found no significant difference in average spine density between the CTL and cKO (Fig. 4F, Spine density per 100 µm dendrite CTL = 94.6 ± 1.8; cKO = 88.9 ± 3.8), *p* = 0.199 Welch’s t-test; N = 12 mice per genotype). However, there was significantly more animal variability in spine density in MCU cKO mice than CTL mice (*p* = 0.0282, F = 4.056.77; F test for variance). Taken together, this confirms that there is no change in spine density in MCU cKO mice compared to CTL mice.

## Discussion

In the present study, we examined the role of MCU in promoting input-specific plasticity in CA2. We found that MCU is necessary for LTP at CA2 distal synapses where MCU expression is enriched, but MCU deletion did not alter the lack of plasticity at CA2 proximal synapses. The LTP deficit at synapses in CA2 distal dendrites of MCU cKO correlated with a decrease in dendritic spine head size compared to CTL distal dendrites without a change in overall spine density. Looking at the effect of MCU cKO on mitochondria ultrastructure in CA2 dendrites, mitochondria were smaller, more numerous and closer together in MCU cKO mice, suggesting there is more mitochondrial fragmentation. More fragmented mitochondria were seen across all dendritic layers of CA2. Accordingly, the loss of MCU did not alter the layer-specific differences in mitochondria morphology across CA2 dendrites, suggesting that the asymmetrical expression of MCU across CA2 dendritic layers is not necessary to establish or maintain the structural diversity of mitochondria. However, MCU enrichment in CA2 SLM may still confer unique functional properties to mitochondria that are necessary for LTP at those synapses. While plasticity at the ECII-CA2 synapse has not yet been functionally linked to social memory, activity at the lateral ECII-CA2 synapse is essential for social memory^49^. Recent evidence links mitochondrial function to social behavior and deficits^50^, however whether MCU-enriched mitochondria in CA2 contribute to its role in social memory remains to be tested.

### A role for MCU in the propensity of CA2 distal synapses to undergo LTP

It was recently shown that MCU is required for action potential evoked production of NADH by mitochondria in acute cortical slices^32^. While these data focus on the role of MCU in powering sustained action potential firing, others have shown mitochondrial calcium uptake in cortical neuron dendrites from acute slices selectively occurs with the coincidence of pre- and postsynaptic activity, suggesting that dendritic mitochondria may respond selectively to plasticity-inducing stimuli^33^. Thus, it is possible that MCU plays a general role in proper LTP expression by boosting ATP, but differential MCU expression may tune the mitochondrial response to the unique calcium dynamics of the cell or circuit. Indeed, high-frequency action potential firing causes MCU-dependent mitochondrial calcium uptake that differs between the hippocampus and cortex^32^, suggesting that variability in the coupling of activity and mitochondrial calcium uptake due to differences in MCU expression could be a mechanism for scaling energy production to meet cell-type specific needs.

Many features of CA2 neurons are unique compared to neighboring hippocampal neurons. For example, CA2 neurons highly express a number of genes that act as molecular brakes on plasticity^39^, and some of those mechanisms involve restricting calcium signaling^51,53^. LTP is highly dependent on calcium influx into the post-synapse and resulting downstream signaling cascades^52^. CA2 neurons have a significantly faster rate of calcium extrusion than CA1 and CA3 neurons, as well as an increased calcium buffering capacity, which mitochondria may contribute to^53^. The same study also showed that CA2 neurons have the lowest endogenous free calcium at rest and that increasing intracellular calcium levels permits LTP at the typically resistant CA2 SR synapses. Because the majority of the identified brakes on CA2 plasticity are not spatially restricted to SR, it is possible that SLM harbors additional mechanisms to overcome these molecular brakes and express LTP. MCU could fulfill this role, as MCU expression is enriched in SLM compared to SR and one proposed function of MCU is to couple neuronal activity to energy metabolism by decoding intracellular calcium levels^32,33,54^. We speculate that the enrichment of MCU in CA2 distal dendrites may promote LTP at ECII-CA2 synapses by enhancing the sensitivity of mitochondria to changes in cytoplasmic calcium, potentially to couple it to ATP production. However, given that alterations in dendritic mitochondria and spine morphology were seen across all dendritic layers in MCU cKO mice, other intrinsic differences in synaptic or circuit properties between proximal and distal dendrites may be contributing to the loss of LTP at distal synapses. In future experiments, MCU could be overexpressed in areas of the hippocampus with low endogenous MCU expression (to avoid causing mitochondrial Ca^2+^-induced neuronal death;^55^), such as in CA1, where overexpressed MCU localizes to proximal dendrites^6^. In theory, MCU overexpression in CA1 neurons might be expected to enhance the expression of LTP at proximal CA1 synapses by boosting ATP production.

Because the CA3-CA2 synapse in SR is resistant to LTP, the lack of an effect of MCU deletion on synaptic plasticity in SR was unsurprising. However, another important function of MCU is to buffer intracellular calcium and one might expect an impact on CA2 synaptic responses at either synapse due to a change in cytoplasmic calcium handling. Indeed, we previously found that acute blockade of MCU with Ru360 in the patch pipette leads to LTD at CA3-CA2 SR synapses in response to an LTP pairing protocol that normally elicits no change at these synapses^34^. In our MCU cKO mice, we occasionally observed LTD in response to a tetanizing stimulus at ECII-CA2 synapses. ECII-CA2 stimulation in cKO mice elicited LTD in 14% of recordings, whereas no LTD was observed after ECII-CA2 stimulation in CTL mice. This could suggest that MCU deletion generally enhances the probability of getting LTD over LTP due to altered calcium dynamics. However, we note that the frequency of observing LTD in response to an LTP stimulus delivered to CA3-CA2 synapses was not different in cKO versus CTL mice, which is in contrast to what was seen in the acute MCU blockade experiments. It is likely that acutely blocking mitochondrial calcium influx pharmacologically could cause transient changes in cytoplasmic calcium levels that are unlikely to be recapitulated with prolonged blockade of mitochondrial calcium uptake resulting from postnatal genetic deletion. It is also possible that MCU may differentially impact plasticity depending on the cell types and forms of plasticity involved. Consistent with this idea, global MCU haploinsufficiency enhanced presynaptic LTP at DG-CA3 synapses in hippocampal slices^56^. Specifically, clearance of presynaptic calcium by mitochondria was reduced in MCU + / − mice, increasing vesicle release probability, despite reduced ATP^56^. This contrasting finding could be explained by differences in the role of cytosolic calcium versus ATP in presynaptic and postsynaptic LTP (but see also^57^), or due to differences in the effects of cell-specific homozygous MCU deletion mice versus global haploinsufficient MCU mice. While emerging evidence supports a role for MCU in regulating energy production and calcium buffering in both pre^58^ and post^33^ synaptic functions, further studies are needed to resolve its impact on specific cell types and circuits as well as the underlying mechanisms linking it to plasticity.

### MCU loss results in changes to spine morphology

We detected a decrease in spine head area in the distal dendrites of MCU cKO mice that could contribute to the deficit in LTP. An average decrease in spine head area likely indicates a decrease in overall synaptic strength^47,59^, which may make potentiation less likely to occur. Interestingly, although the deficit in LTP was observed at distal synapses, the decrease in spine head area was seen equivalently across all dendritic layers of cKO CA2. These data indicate that MCU deletion does not preferentially impair spines in CA2 distal dendrites, where MCU is enriched. Since synapses in SR are generally resistant to high frequency stimulation-induced LTP, we were unable to determine what effect this would have on this form of LTP at CA3-CA2 synapses. Thus, it is possible that the plasticity deficit is not limited to the distal dendrites. Although we observed a change in the average size of dendritic spines, there was no change in spine density in SLM of cKO mice. Because the majority of these spine heads have a PSD and are opposed to a presynaptic bouton with clear presynaptic vesicles (see Fig. 4A), this suggests that the number of synapses is similar in CA2 SLM of MCU cKO and CTL. Consistent with this, we saw no difference in the amplitude of baseline evoked synaptic responses for any stimulus intensity in MCU cKO compared to CTL at ECII-CA2 synapses (Fig. 2B–C), suggesting that the connectivity of this circuit is not altered by the loss of MCU in CA2 neurons. While we did find changes in dendritic spine morphology, the LTP deficit may also be due to non-structural changes in dendritic spines that would not be captured by our methods. Spines in CA2 SLM receive input from ECII, and activity from the lateral ECII to CA2 is required for social recognition memory (Lopez-Rojas et al. 2021). Given the LTP and spine deficits we uncovered in SLM, further studies are warranted to test whether MCU cKO in CA2 has an effect on social recognition memory.

### Mitochondrial fragmentation due to MCU deletion

Mitochondria are highly dynamic organelles that are shaped by two opposing forces: fission and fusion. Mitochondrial fission, the division of mitochondria to make new or recycle damaged mitochondria, is mediated by the phosphorylation of dynamin-related protein 1 (DRP1), whereas fusion of mitochondria is mediated by optic atrophy 1 (OPA1) and mitofusins (MFN1 and MFN2)^2,61^. The balance of these forces determines mitochondrial form, which is intimately linked to bioenergetics. For example, it’s been shown that larger axonal mitochondria in cortical neurons can produce more ATP^5^. Fragmented mitochondria, which could be due to either less fusion or more fission^60,61^, could result in functional consequences, such as decreased ATP production, altered ROS generation or calcium-induced calcium release, that might underlie a plasticity deficit. There are multiple potential causes of mitochondrial fragmentation after MCU deletion. In cultured rat hippocampal dendrites, mitochondrial fission is rapidly triggered by chemical LTP^22^. This mitochondrial fission requires CaMKII and DRP1 and precedes mitochondrial calcium uptake^22^. Expression of dominant negative forms of DRP1 blocks mitochondrial fission and LTP in both culture and CA1 of acute hippocampal slices^22^. Therefore, loss of MCU may elevate cytoplasmic calcium levels and lead to aberrant activation of DRP1 that could, in theory, result in more fragmented mitochondria if fission can occur without mitochondrial calcium uptake. The effect of MCU loss on mitochondrial number was greatest in SLM, suggesting the mitochondria might be more fragmented in SLM relative to SO or SR in MCU cKO mice—although, it did not scale proportionally with a greater decrease in individual mitochondria area or diameter in SLM.

On the other hand, inhibition of MCU has been shown to prevent mitochondrial fission and fragmentation in cultured hippocampal neurons during ischemia^29^. MCU has also been shown to mediate mitochondrial fission in rat cortex^30^. Although these studies are in contrast to our findings that loss of MCU results in an increase in mitochondria fragmentation in CA2, most have looked at MCU loss in the context of injury and disease. Endogenous MCU expression is also highly variable across cell types, thus different cell types may have different sensitivities to MCU-induced mitochondrial fragmentation and cell death^55^. It is also possible that the fragmentation we found is not due to increased fission mediated by DRP1, but instead an impairment in fusion, or a result of unhealthy or damaged mitochondria. Lewis et al. showed that a loss of synapses correlated with increased local mitochondrial fission and ULK2-dependent mitophagy in CA1 apical dendrites in an Alzheimer’s disease model^62^. However, we did not observe a loss of mitochondrial biomass and the mitochondria in our MCU cKO mice appear ultrastructurally normal in electron micrographs. Even so, the mitochondrial structural changes we observed (i.e. fragmentation) may indicate functional changes that would not necessarily be observed in electron micrographs.

### MCU deletion alters the dendritic mitochondrial network

Generally, the spatial distribution of mitochondria strongly correlates with predicted energy usage, which in neurons is highest at the synapse^63^. In this study and our previous study, we found that CA2 distal dendrites (SLM) harbor more mitochondrial mass than proximal (SR) and basal (SO) dendrites, suggesting that synapses in distal dendrites require more energy. We reasoned that this might be due to the propensity of CA2 SLM synapses to undergo LTP. However, in MCU cKO mice that fail to produce robust LTP at SLM synapses, the relative increased mitochondrial mass in SLM remains. Indeed, the structural heterogeneity across dendritic layers in MCU cKO was similar to CTL, except that mitochondria were overall smaller and more numerous across all dendritic layers in MCU cKO mice (Fig. 3 and Supplemental Fig. 4). Interestingly, mitochondrial length did not decrease in SLM as it did in SO and SR of cKO mice. However, the relative ultrastructural differences across dendritic layers were unchanged. This indicates that MCU expression is not related to the differences in mitochondrial structure across CA2 dendrites. Similar heterogeneity in mitochondrial structure has also been reported in CA1 neurons, with CA1 SLM harboring larger mitochondria than CA1 SR^64^, suggesting this may be a general phenomenon in hippocampal pyramidal neurons. Virga et al. showed that the difference in mitochondria shape across CA1 dendritic compartments is activity-dependent and regulated by layer-specific differences in AMPK and CAMKK2, which regulate fission/fusion factors. Increasing neuronal activity with picrotoxin induced mitochondrial fission in CA1 dendrites, while acute silencing of CA1 neurons led to an elongation of mitochondria throughout CA1 dendrites. This is in contrast to our findings in CA2, where CA2 SLM has larger mitochondria than SR, despite having a higher synaptic drive and propensity for plasticity relative to CA2 SR^40^. This discrepancy could be due to a difference in the balance of fission/fusion factors and/or differences in calcium dynamics between CA1 and CA2 neurons, highlighting that mitochondrial morphology is differentially regulated in different cell-types and circuits. Further studies will be needed to directly compare mitochondrial structure and the expression of fission/fusion factors across CA2 and CA1 dendrites.

The spatial distribution of mitochondria throughout the dendrites can be assessed by metrics such as the number of mitochondria and the distance between them. We found that the distances between neighboring mitochondria were reduced in MCU cKO mice across CA2 dendrites, suggesting that the distribution of the mitochondrial network may be altered. The distribution of mitochondria in dendrites is critical for synaptic function^17^. While mitochondria are rarely seen inside dendritic spines in mature neurons, spines are seen to contain endoplasmic reticulum (ER) associated with nearby mitochondria in electron micrographs from CA1, CA3, and DG of ground squirrels^65^. Mitochondria are known to form close connections with ER (< 200 nm) at zones called “mitochondrial associated membranes” (MAM), which allow for communication and the transfer of proteins, ions and metabolites between mitochondria and ER^27,66^. It is thought that calcium release from the ER at MAMs is taken up into the mitochondria via MCU and voltage-dependent anion channels on the outer mitochondrial membrane^27^. MCU deletion could potentially alter these MAM domains and disrupt the ER-mitochondria connection, which would likely have functional consequences at the synapse^67^. This could be one possible explanation for the altered distribution of mitochondria we found in MCU cKO mice, including a decrease in nearest neighbor distance and a “stacked” orientation of the mitochondria in some dendrites. The resolution was not high enough in our SEM dataset to segment or quantify ER-Mitochondria contacts. Mitochondria are also seen to form filamentous reticular networks between other mitochondria in dendrites from CA1, CA3 and dentate gyrus^65^, which could also be disrupted by MCU cKO. While this has not been studied in CA2 of the hippocampus, we observed what appear to be thin connections between dendritic mitochondria at both the electron microscopy and immunohistological level.

### Limitations

We acknowledge that in the present study we have shown correlations, but not direct causal relationships, between changes in mitochondria morphology and LTP or spine changes. It’s possible these effects occur by different, potentially independent, or indirect mechanisms. LTP phenotypes can be caused by changes in neuronal excitability, however MCU loss in CA2 did not alter baseline synaptic activity at proximal or distal synapses (Fig. 2B-C), and there was no difference in cFOS expression in CA2 neurons in cKO mice compared to CTL, (Supplemental Fig. 6; *P* = 0.70, two-tailed unpaired t-test). Further studies will be needed to determine whether MCU deletion in CA2 neurons impairs plasticity through compromised mitochondrial metabolism; however, this is technically difficult to measure in situ in such a small subregion in a layer specific manner. It is also an open question whether our results are CA2-specific or could be generalizable to other brain areas. In addition, because the cre-dependent recombination of MCU occurs sometime between the age of postnatal (p)4–14, and it takes several weeks to lose MCU expression, it is unlikely for developmental effects to contribute to the LTP phenotype, however, there is the potential for compensation to occur. Compensation could involve MCU-independent methods of calcium entry into the mitochondria, or a downregulation of calcium efflux. MCU-independent calcium influx has been observed in astrocytic mitochondria^68^, however, given that MCU loss or inhibition by Ru360 blocks calcium uptake in mitochondria from heart, liver, and neurons^69,70^, this is unlikely to be the case. Compensation could also involve a shift in mitochondrial respiration to rely more on the malate-aspartate shuttle (MAS). Studies show there is a reversible inhibition of MAS by MCU activation, caused by increased calcium levels in the mitochondrial matrix of isolated mitochondria from brain, liver, and heart^71,72^, suggesting that a metabolic switch could be made to compensate when mitochondrial calcium levels are low. However, this would not explain a deficit in LTP after MCU deletion.

Another potential limitation is that mitochondria ultrastructure in 2D SEM images do not provide a full picture of the volume and shape of mitochondria in 3D. The mitochondria objects measured likely do not represent individual mitochondria, but rather mitochondrial segments, potentially from the same mitochondrion. Thus, mitochondrial count reported here is likely an overestimation of the true count, and metrics such as mitochondrial area and length are likely an underestimation of a full mitochondrion in 3D. This could particularly impact metrics in CA2 SLM, where mitochondria are longer and there is more dendritic branching, both of which could result in individual mitochondria going in/out of plane more frequently and being counted as multiple mitochondria. Additionally, due to the systematic sampling of non-overlapping tiles (8.2 µm × 12.2 µm), the longest continuous dendritic segment measured was 14.3 µm, which limits the maximum Feret’s diameter we can detect. However, these limitations apply across all groups, thus we can detect relative changes in average Feret’s diameter, count and area across layers and genotypes.

In addition, the AI did occasionally miss dendritic mitochondria (~ 1 dendritic mitochondrion missed per 100 µm^2^) and, less commonly, segment non-dendritic mitochondria (2.6% of segmented mitochondria). Importantly, the AI performed similarly across cKO and CTL images (Error rate per 100 µm^2^: 2 in cKO; 1.7 in CTL), which allows us to confidently compare dendritic mitochondria across cKO and CTL mice. In addition, the AI analysis replicated what has been previously shown in wild-type CA2 SR and SLM with a genetic fluorescent mitochondrial tag as well as with manual segmentation of SEM images^6^. However, in SO, we found a larger median mitochondrial area relative to SR and SLM in CTL CA2 using the AI segmented mitochondria compared to the manual segmented mitochondria. Importantly, the manual segmentation included all mitochondria within an ROI (axonal, dendritic, glial) not just dendritic mitochondria, and it was performed on a small subset of images (different ROIs) from the same SEM dataset. To confirm the difference in mitochondria area between the manual and AI analyses, we ran the AI on the same subset of ROIs that were manually analyzed and found a larger average dendritic mitochondria area in SO relative to SR and SLM. However, the average dendritic mitochondria area increased for all layers compared to the manual analysis, just not to the same degree. The increase in mitochondria area was the greatest in SO and the least in SLM (AI mean / Manual mean, SO: 2.3, SR: 2.1, SLM: 1.8). Thus, the difference in mitochondria area between the AI and the manual analysis appears to be due to the smaller fraction of dendritic mitochondria in the SO ROIs compared to SR and SLM (fraction of AI detected dendritic mitochondria / manually detected total mitochondria; SO: 0.19, SR: 0.24, SLM: 0.31). Moreover, the relative differences in Feret’s diameter and count across layers were maintained as seen with the manual and full AI dataset (Avg. Feret’s diameter subsetted AI; SO: 0.95 + − 0.87, SR: 1.17 + − 1.4, SLM: 1.21 + − 1.4; see Table 1 for the full dataset). Thus, SO dendritic mitochondria are similar in size compared to SLM dendritic mitochondria but more round in shape and fewer in number. Others have made comparisons of pre- and postsynaptic 3D mitochondrial ultrastructure in SEM across nucleus accumbens (NA), CA1, cortex and dorsal cochlear nucleus (DCN)^73^. Consistent with these results, we qualitatively observed that dendritic mitochondria were large and filamentous compared to axonal mitochondria in our 2D SEM dataset (Supplemental Fig. 2A). It is important to emphasize that traditional statistical measurements overestimate the significance of detected effects by treating individual mitochondria as statistically independent. Other EM studies similarly analyze mitochondrial ultrastructure at the level of individual mitochondria^4,18^, however, they typically do not have thousands of mitochondria per sample. To account for this limitation, we included a hierarchical statistical bootstrap to interpret the results in terms of Bayesian probabilities for the quantification of mitochondria and dendritic spines in the SEM dataset (Supplemental Figs. 3–5). Generating repeated samples of the data with a bootstrap provides an estimate of the variability and uncertainty in the observed data, which boosts confidence in the robustness of the results.

Combined, our results demonstrate a role for MCU in modulating plasticity in CA2 distal dendrites and for maintaining proper spine and mitochondrial morphology and distribution in dendrites, but MCU is dispensable for mitochondrial structural diversity across CA2 dendrites. We speculate that MCU may generally function postsynaptically to decode cytoplasmic calcium signals to boost metabolic output leading to long lasting changes in synaptic efficacy and that differences in postsynaptic MCU expression may reflect a general mechanism to tune ATP production in different calcium contexts.

## Methods

All procedures were approved by the Institutional Animal Care and Use Committee of Virginia Tech and carried out in accordance with their regulations. Methods are reported in accordance with ARRIVE guidelines.

### Animals

All experiments were performed on adult (8–16 week old unless otherwise noted) male and female mice on a C57BL/6 J background sourced from Jackson Laboratory and NIEHS. MCU CTL and cKO mice were generated by crossing a CA2-specific Amigo2-cre mouse line (NIEHS, B6(SJL)Tg(Amigo2-Cre)8Ehs^41^ *BioRxiv,* Figure S13–S17) to a floxed MCU line (MCU^fl/fl^, B6;129S-Mcu^tm1.1Jmol/J; Jax Stock No. 029817^42^). Resulting heterozygous mice were crossed to produce MCU^fl/fl^;Amigo2-cre positive and negative mice, which were then bred together to produce the experimental animals. Genotypes were confirmed for the MCU floxed or WT allele and the presence or absence of cre using Transnetyx genotyping service. The Amigo2-cre line has been validated for conditional deletion of knocked in floxed alleles, demonstrating cre recombination occurring between postnatal ages p4 and p14^74^. Mice were group-housed when possible under a 12:12 light/dark cycle with temperature and humidity control and access to food and water ad libitum. Experimental mice were euthanized with an IP injection of Sodium Pentobarbital (100 mg/kg) unless otherwise specified in methods.

### Electrophysiology: in vitro brain slice preparation and recording

Experiments were performed on litters at ages 10–20 weeks with the experimenter blind to genotype. Cutting and recording solutions were made as described in^75^. Mice were deeply anesthetized with 4% isoflurane and decapitated. The brain was rapidly removed and cooled for 2 min in ice-cold cutting solution containing (in mM): 10 NaCl, 2.5 KCl, 10 D-( +)-glucose, 25 NaHCO_3_, 1.25 NaH_2_PO_4_, 2 sodium pyruvate, 195 sucrose, 7 MgCl_2_, 0.5 CaCl_2_, and saturated with 95% O_2_/5% CO_2_ with a final pH of 7.4. Horizontal slices of the hippocampus were cut at 300 µm using a vibratome (VT1000S, Lecia) and placed in artificial cerebrospinal fluid (ACSF) containing (in mM): 125 NaCl, 2.5 KCl, 1.25 NaH_2_PO_4_, 25 NaHCO_3_, 20 D-( +)-glucose, 2 Na-pyruvate, 2 MgCl_2_, 2 CaCl_2_, and saturated with 95% O_2_/5% CO_2_ with a final pH of 7.4. Slices were incubated in ACSF at 33 ± 1 °C for 20 min and then at room temperature for > 40 min prior to recording.

For recording, slices were transferred to a submerged recording chamber perfused continuously with 3 ml/min of oxygenated ACSF at 33 ± 1 °C. CA2 pyramidal neurons were visualized using a Zeiss microscope (Axio Examiner.D1; Zeiss) equipped with a W Plan-Apochromat 40 × water immersion lens configured for DODT gradient contrast (DGC) microscopy. A stimulating electrode (model #30213; FHC Inc.) was placed in either the SC to stimulate inputs to the SR, or PP to stimulate inputs to the SLM. For recording, glass micropipettes (O.D. 1.5 mm, I.D. 1.12 mm; A–M Systems) were pulled on a vertical puller (PC-10, Narishige) to make field potential (FP) pipettes (1.2 ± 0.5 MΩ). FP pipettes were filled with ACSF and placed in the stratum pyramidal of CA2. CFDA-SE (an amine-reactive cell-permeable fluorescent green dye; Thermo-Fisher) was added to the pipette solution and pressure ejected after recording was completed to allow post hoc identification of the recording site (only recordings made from CA2 were kept for analysis).

Afferent stimulation consisted of constant current square wave pulses at 50–450 µA (set to 50% of maximal FP response) and 100 µsec in duration. Pre-conditioning baseline recordings of evoked FP peak amplitudes were made at a stimulation frequency of 0.1 Hz for 10 min. This was followed by a 20-min period of synaptic conditioning, consisting of three stimulus trains of 100 Hz for 1 s, interleaved with two 10 min rest periods without stimulation^76^,“STET” protocol). Finally, post-conditioning evoked FP peak amplitudes were evaluated at 0.1 Hz for a period of 60 min.

All recordings were made with a MultiClamp 700B amplifier, digitized at 20 kHz with a Digidata 1440A and recorded using Clampex 10.7 software (Axon Instruments, Molecular Devices). Recordings of evoked FP peak amplitudes were measured as the difference between baseline and peak (analyzed with a 1 ms smoothing window). Any recordings with an unstable baseline (linear best fit of all preconditioning FP peak amplitudes had an r^2^ > 0.2) were discarded. Only one recording was made from each slice, so that a single stimulation protocol was applied in each case.

Data was analyzed using Clampfit 10.7 software (Axon Instruments, Molecular Devices). We assessed the plasticity of the FP response in each slice by calculating a post/pre ratio (the average FP peak amplitude for the last 5 min of post-conditioning divided by the average FP peak amplitude for the last 5 min of pre-conditioning). We defined LTD as a significant decrease (> 10%) in post/pre ratio, LTP as a significant increase (> 10%) in post/pre ratio; or no change (not significantly different, or < 10% change) in post/pre ratio (*p* < 0.05, *t*-test). Data displayed as time plots show values for normalized FP peak amplitudes averaged over 1-min intervals (i.e. each data point represents the average of 6 data points collected at 0.1 Hz).

Post Hoc immunofluorescence staining was used to validate the recording site in area CA2^75^. After recording, slices were post-fixed in 4% paraformaldehyde for 12–48 h then transferred to 1X PBS. Slices were permeabilized and blocked overnight with 3% Normal Goat Serum (NGS) in 1X PBS-0.3% Triton X-100 (0.3% PBST) before 2–3 day incubation with primary antibodies at 4C for PCP4 (1:250, Invitrogen Cat# PA5-52,209, RRID:AB_2645298) or NECAB2 (1:250, Novus Cat# NBP1-84,002, RRIS:AB_11028373). Slices were washed in 0.3% PBST several times and then incubated with AlexaFluor goat anti rabbit 633 (1:250, Sigma Cat# SAB4600141) overnight. After washes with 0.3% PBST, slices were stained with DAPI (Sigma Aldrich D9542, 1:10,000 in PBS) incubated for 30 min in 60% TDE prior to imaging cleared slices weighted with harps in a 6 well glass bottom plate (Cellvis P06-1.5H-N) on a Leica Thunder microscope at 20X.

### Immunofluorescence

Mice were anesthetized with 150 mg/kg sodium pentobarbital and transcardially perfused with 15–20 ml of ice-cold 4% paraformaldehyde. Brains were dissected and post-fixed for at least 24 h before sectioning 40 μm thick sections in the horizontal plane on a vibratome (Leica VT1000S). All brain sections immunostained with MCU were washed in 1X PBS-0.1% Triton X-100 (0.1% PBST) before they underwent antigen retrieval by boiling free floating sections for 5 min in 1 ml of nanopure water, followed by a permeabilization step in 0.1% PBST for 15 min at room temperature. All sections were then blocked for 1 h in 5% Normal Goat Serum (NGS) in 0.1% PBST. Sections were incubated overnight at 4C (18–24 h) with primary antibodies: rabbit-anti-MCU (1:2000, Novus Cat# NBP2-76,948, Lot# H660681004, RRID:AB_2924913) and mouse-anti-RGS14 (1:500, NeuroMab Cat# 75–170, RRID:AB_2877352) or mouse-anti-cFOS (1:4000, Santa Cruz Bio Cat# sc-271243, RRID: AB_10610067) and guinea pig-anti-PCP4 (1:2000, Synaptic Systems, Cat# 480 004, RRID: AB_2927387). Sections were then washed in 0.1% PBST several times and blocked for 30 min in 5% NGS in 0.1% PBST. Sections were incubated for 2 h at room temperature in secondary antibodies (1:500, Invitrogen, AlexaFluors goat anti rabbit 546 Cat# A11035, goat anti mouse 488 Cat# A11029, goat anti mouse 546 Cat# A11030, goat anti guinea pig 488 Cat# A11073) followed by several washes in 0.1% PBST and a final wash in 1X PBS.

### cKO validation

Figure 1 includes MCU histology data from 5 to 6 mice aged 8–17 weeks and 2 mice aged 32–62 weeks per genotype. The results from older mice did not significantly differ from younger mice. 20 × (0.8 NA) 16-bit images of MCU and RGS14 immunolabeling were acquired on a Leica Thunder epifluorescence microscope (Leica DMi 8) using identical acquisition parameters for CTL and cKO and Lightning computationally clearing. MCU fluorescence was quantified in FIJI (v. 2.1.0/1.53c, NIH)^77^ using max projected images from 3 to 5 sections per animal. CA2 neurons were identified via RGS14 labeling. A line ROI was drawn along the length of CA2 neurons (line length 650 µm, drawn at the end of the mossy fiber track with the start of the line being in SO and the midpoint of the line at the middle of SR), CA1 neurons (line length 700 µm, starting at SO with the midpoint of the line at the middle of SR), and DG granule neurons (line length 300 µm, starting at the granule cell body layer, with up to 75 µm covering the cell body layer when possible and the rest of the line on the molecular layer). Fluorescence values along the line were obtained using the FIJI Analyze and Plot Profile functions. For the neighboring cortex, a 400 µm × 400 µm ROI was cropped out of the original image and fluorescence intensity was measured using the Measure function in Fiji. Fluorescence background noise was subtracted for all regions using the negative control (no primary antibody) sections. Due to differences in the length of the CA regions along the dorsal–ventral axis, some of the ROI lines yielded zero values from the line being beyond the image border. These values were removed before averaging across sections per animal. The data were then binned by 10 microns length and averaged across sections to obtain one average fluorescence by distance line plot per animal. The data were then normalized to the CTL animals run in the same IHC cohort. In order to compare across subregions, the peak binned value representing the cell body layer per section was averaged across sections per animal, and compared across regions such that every animal is represented in each region. MCU cKO was further validated through quantification of CA2 cell count using the Cell Counter tool in Fiji. An equal z-stack size was used for all images and cell bodies that expressed MCU and/or RGS14 were manually counted in each of these z sections. Experimenters were blind to genotype through the analyses and used RGS14 to guide placement of lines and cell counts. However, due to the obvious deletion of MCU expression in CA2 neurons, experimental bias could not be completely eliminated.

For the quantification of cFOS positive cells in CA2 across genotypes (Supplemental Fig. 6), 16 bit z-stack images were acquired on a Leica Thunder at 20X (0.8 NA) and the resulting images were set to a fluorescence range of 100–400 and max projected prior to quantification. cFOS + cells were identified using the LUT “spectrum” and defined as any cells with nuclear cFOS labeling at a fluorescence intensity > 250 grey value (light blue and above on the intensity color map) that also were PCP4 + . Cell counting was done manually (blind to genotype) with the multi-point tool in Fiji, and the percentage of cells positive for cFOS and PCP4 labeling was normalized by the total number of PCP4 + cells in each image. 4–6 sections were analyzed per mouse for 5 CTL and 6 cKO mice.

### Protein-retention expansion microscopy (ProExM)

40 μm horizontal mouse brain sections were immunostained then expanded with 4 × protein expansion microscopy (ProExM) as previously described in^78^ using an *Amigo2*-EGFP line to selectively label CA2. Briefly, sections were treated as described above with the following modification. All sections were washed in PBS and blocked for at least 1 h in 5% Normal Goat Serum (NGS)/0.3% Triton-100x. Antibodies (MCU, 1:500 and Chicken anti-GFP, 1:500 Abcam ab13970) were diluted in blocking solution and sections were incubated for 72 + hours at room temperature (RT). After several rinses in PBS-T (0.3% Triton-100x), sections were incubated in secondary antibodies (1:500, Invitrogen AlexaFluors, goat anti chicken 488 Cat# A11039 and goat anti rabbit 546 Cat#A11035) for 48 h at RT. Prior to imaging, adjacent unexpanded sections that were run simultaneously with expanded sections were washed in PBS-T and mounted under Vectashield fluorescence media to calculate pre-expansion nuclei diameters.

Sections to be expanded were incubated overnight in Acryloyl-X stock/PBS (1:100, ThermoFisher, A20770) at room temperature in the dark. All solutions were prepared as described by^79^. Following incubation, the slices were washed twice with PBS for 15 min each at room temperature. The gelation solution was prepared by adding 384 uL of monomer solution, 8 uL 4-Hydroxy-TEMPO inhibitor (1:200 w/v, Sigma Aldrich, 176141), 8uL TEMED accelerator (10% v/v, Sigma Aldrich, T7024), and lastly 8uL of APS initiator (10% w/v, Sigma Aldrich, 248614) for each section. Sections were incubated in the gelation solution for 30 min at 4C in the dark. Gelated sections were placed on gelation chambers constructed from microscope slides with coverslips as spacers. Our gelation chambers produce gels with the thickness of a single No. 1.5 coverslip (~ 0.15 mm thick). The chambers were filled with gelation solution and allowed to incubate at 37C for 2 h in a humidified container. Following gelation, the gelation chamber was deconstructed and the gel was carefully removed from the chamber using a coverslip and Proteinase K-free digestion solution. Gels were then placed in digestion solution containing proteinase K (8U/mL, New England BioLabs, P8107S) and digested for 4 h at room temperature.

Gels were stained with DAPI (Sigma Aldrich, D9542; 1:10,000 in PBS) for 30 min at room temperature with shaking. Finally, the gels were washed twice with PBS for at least 20 min and either stored in PBS at 4C or fully expanded in npH20 for imaging. Images of CA2 SLM dendrites were acquired using 4X Super Resolution by Optical Pixel Reassignment (SoRa) on an inverted spinning disk confocal microscope (Yokogawa CSU-W1 SoRa/Nikon Ti2-E Microscope) equipped with Hamamatsu Fusion BT cameras, and 20X water (0.95 NA. WD 1.0 mm) or 60X oil (1.49 NA. WD 0.14 mm) immersion lenses.

### Scanning electron microscopy

This protocol was adapted from the protocol version 2 published by NCMIR at UCSD (Deerinck et al. 2022). Mice were anesthetized with sodium pentobarbital (euthanasia solution, 150 mg/kg) and perfused with ice-cold 0.1 M cacodylate buffer pH 7.4 containing 2.5% glutaraldehyde, 2% paraformaldehyde with 2 mM calcium chloride for 3 min. The brain was removed and fixed overnight at 4C in the same fixative before vibratome sectioning (Leica VT1000S) into 350-micron thick sections in the 0.1 M cacodylate buffer pH 7.4 with 2 mM calcium chloride. Sections were placed back in fixative for microdissection three days later. Hemisected brain sections were placed on wax paper with a drop of fixative and a 2 mm × 2 mm hippocampal microdissection was obtained per brain and placed back in fixative for further processing. Tissues were postfixed with 1.5% potassium ferrocyanide plus 2% osmium tetroxide then en bloc stained with incubations in thiocarbohydrazide solution, osmium tetroxide, uranyl acetate, and Walton’s lead aspartate. Dehydration was performed by an ethanol gradient and finished in propylene oxide. Tissues were embedded in Epon 812. The embedded tissues were sectioned to 120 nm (Leica EM UC7 ultramicrotome), mounted on a silicon wafer, and imaged in a ThermoFisher Aprea Volumescope at 2 nm pixel size. Three 150 × 150 µm regions of interest were obtained per section (basal, proximal, distal CA2 dendrites) from MCU cKO and CTL mice. A representative EM hippocampal section with ROIs drawn is shown in Fig. 3A.

### Analysis of SEM images with Biodock

Regions of interest (ROIs, 150 × 150 µm) in SO, SR and SLM of CA2 in three 120 nm sections from each of three CTL and cKO mice were analyzed. A subset of the CTL data from these animals was previously published^6^, but was reanalyzed with the AI to allow direct comparison with the cKO and validate the performance of the AI. The larger ROIs were acquired as 14 × 20 100 µm^2^ tiles with 10% overlap for stitching (~ 12.2 × 8.2 µm). Every 8th tile was selected for analysis to avoid analyzing overlapping tiles. A similar sampling scheme was used for a handful of images that were acquired as 2 × 2 6,700 µm^2^ tiles with 10% overlap, resulting in the same-sized 100 µm^2^ tiles for analysis. Image sampling and processing were done in batches with a custom Python code. Images were inverted if necessary, and a Gaussian blur with a radius of 2 nm was applied to all EM images for training and analysis. Tiles were excluded from analysis if they had poor image quality, significant artifacts or rips, contained only axonal tracts or cell bodies, or were otherwise unfit to analyze. A total of 1559 tiles (~ 156,000 µm^2^) were analyzed across the 6 mice.

Biodock AI was used to segment dendritic mitochondria^80^. To train the AI, we separated a subset of data (~ 1% of the total dataset) for training, making sure to include training data from each layer and genotype in the analysis, as well as undesired elements such as cell bodies, artifacts and blank spaces. We selected an appropriate tile size in Biodock (4000 × 4000 pixels) and labeled all dendritic mitochondria in the training images as an object type class. To be counted as dendritic mitochondria, the object must have distinct borders, a solid dark fill, and be located inside a clear dendrite segment not bordered by myelin or containing synaptic vesicles. We excluded fragments of mitochondria at image edges or if the identity or location of the object was ambiguous. We proceeded to train the AI on the dendritic mitochondria object class, allowing augmentations such as image flipping and brightness/contrast adjustments. We assessed the AI’s performance with a manual spot check on a test dataset of about 2% of the final dataset. The average number of mistakes per tile was counted by category. Categories included missing dendritic mitochondria, border errors, object merging, detection of axonal or soma mitochondria, and identification of non-mitochondrial elements like myelin. The overall sensitivity of the AI model in correcting identifying dendritic mitochondria was 92.2%.

The trained AI was then used to analyze mitochondrial morphology in the final dataset of 3 CTL and 3 cKO mice. We configured the AI project settings to define the analysis metrics of interest and set a confidence threshold of 0.4. A size threshold was applied to exclude mitochondrial objects smaller than 0.01 µm^2^ or larger than 2.1 µm^2^, which reflects the minimum and maximum area of visually confirmed mitochondria objects in the dataset. A total of 23 objects out of 23,682 were excluded with the size threshold, the majority of which were a result of segmentation errors. All pixel measurements were converted to microns for length and area, and the aspect ratio was calculated by dividing the short axis by the long axis of each object. Maximum Feret’s diameter was computed as the longest distance between points around an object’s convex hull contour. Total mitochondrial count and total mitochondrial area were summed per 100 µm^2^ tile, and a nearest neighbor analysis was performed with the KDTree function in the Python package Scipy^81^ to determine mitochondrial Euclidean distances. Data for the plots in Fig. 3 were normalized to the combined mean of the control. Bayesian statistics were applied (described below) on individual mitochondria area and mitochondrial count per 100 µm^2^.

A separate Biodock AI was trained to segment dendritic spine heads in a subset of the same SEM dataset where synapses could be resolved. To be counted as a dendritic spine, the criteria was a rounded membrane bound structure with a clear post-synaptic density and no pre-synaptic vesicles. The majority of dendritic spines were opposed to a presynaptic bouton with vesicles; however, this was not a requirement. For ease of segmentation, dendritic spine heads attached to a dendrite in cross-section were excluded from the dendritic spine analysis. A spot check of about 1% of the dataset confirmed that the AI correctly detected about 97% of spines in both CTL and cKO. The trained AI was used to segment dendritic spine heads in the subsetted dataset, which had a combined area of 34,800 µm^2^ from 2 CTL and 52,700 µm^2^ from 3 cKO mice, using the same image tile sampling and processing as described above and a confidence threshold of 0.7. Custom Python code was used to parse the segmented object data, convert pixels to microns and sum the number of dendritic spine heads per 100 µm^2^ image tile. The same hierarchical bootstrap was done on dendritic spine head area and dendritic spine head count per 100 µm^2^.

### Hierarchical statistical bootstrap of mitochondria and dendritic spine ultrastructure

A hierarchical bootstrap was performed in Python to compare individual mitochondria area and mitochondria count per image tile across dendritic layers in the cKO and CTL. Our hierarchical bootstrap was modeled after^43^. For mitochondria count per tile, the SEM data was grouped by genotype and 3 animals from each genotype were randomly sampled with replacement. From those resampled animals, 3 hippocampal sections were randomly sampled with replacement from each animal. To determine the ideal sampling size for image tiles, the bootstrap was run multiple times to randomly sample either 20 tiles (undersampling), 32 tiles (max number of tiles per group), or 50 tiles (oversampling) from each sampled hippocampal section. For each iteration in the bootstrap (n = 1000 for the sampling tests), the median mitochondria count per tile within each group was calculated. Oversampling the tiles reduced the bootstrap variability across trials,thus, for the final bootstrap, 50 tiles were randomly sampled per hippocampal section (n = 450 tiles per group). In general, oversampling the data with replacement in a bootstrap analysis provides a better approximation of the original population’s distribution.

For the mitochondria area, we went on to randomly sample the individual mitochondria objects from each sampled image tile to get the median of the resampled population of mitochondria. Another systematic sampling test was done to determine the ideal number of mitochondria to sample from each tile. The bootstrap was run with a sampling size of either 5, 10, 20, 50 or 100 mitochondria from each of the 50 resampled image tiles. It was determined that 100 mitochondria was the most appropriate sampling size, so the final bootstrap sampled 100 mitochondria from each of 50 sampled tiles to get the medians for each layer (n = 45,000 mitochondria per group). For more robust results, the final bootstrap was run on mitochondria count and mitochondria area with 10,000 iterations instead of 1000. A custom Python code was used to plot the distribution of the resampled medians comparing the bootstrap population across layers and genotypes (Supplemental Fig. 3). For each probability distribution plot, the medians were binned in linear space into 10 equal bins (for count) or 100 equal bins (for area). For the comparisons of interest, stacked bar plots were also created showing the proportion of bootstrap iterations where the median for each group in the comparison was greater than the second group.

The same bootstrap was performed for spine head area and number of spine heads with 10,000 iterations. The data was resampled at the level of animal and hippocampal section with a sampling size equal to the N of animals or stubs per animal (i.e. animal sample size was 2 for CTL and 3 for cKO based on the number of animals). For each of the resampled stubs, 50 tiles were sampled and the median of the resampled population was calculated for the tile level data (number of spine heads per tile). For individual spine head area, 100 spine heads were further sampled from each tile to generate a resampled median spine head area. Custom Python code was then used to generate probability distribution plots and compare the bootstrap population across layers and genotypes, similar to the analysis of segmented mitochondria (Supplemental Fig. 5).

### Golgi staining

Mice aged 8–16 weeks were perfused and postfixed for at least 24 h with 2.5% glutaraldehyde/2% paraformaldehyde. Then, brains were dissected in 5 mm^3^ blocks and stained with FD Rapid GolgiStain™ Kit (CAT# PK401) as described in the product manual. A subset of the mice used for Golgi analysis were also used for SEM and processed as 350 µm thick slices as described above. Thick brain slices were wrapped with uniformly thicker 2 mm brain sections and processed as tissue bundles as described in Harris et al.^82^ for Golgi-Cox impregnation using the same kit. Both 5 mm^3^ blocks and 350 µm slices were cryoprotected in 30% sucrose for 4–5 days until they sank. Then they were blocked in OCT and cryosectioned at 40 µm thick in the horizontal plane using Leica Cryostat (Leica CM1860). CTL (n = 12) and cKO (n = 12) brains were processed in pairs containing each genotype to avoid any potential batch issues. High resolution 63X (NA 1.4) images of CA2 SLM (up to 25 μm total on Z-axis, optical section thickness = 0.21 μm) were acquired using brightfield microscopy on a Leica Thunder microscope. The CA2 region was identified by a larger cell body size compared to CA1, absence of thorny excrescences on the proximal apical dendrites, diagonal location from the upper lip of dentate, and the abrupt termination of mossy fiber pathways in CA2/CA1 cell bodies borders. CA2 apical dendrites were followed from the cell body to identify branches in SLM and individual dendrite segments in the plane were cropped from the 63X images using Fiji and randomly chosen for analysis. 12 dendritic segments were analyzed from 3 to 5 sections per mouse for each genotype. Fiji plugin “Dendritic spine counter” was used to measure dendrite length, total spine count and spine density (number of spines/length of the dendrite) from the cropped dendrites with manual adjustments where necessary^83^. In summary, ~ 250 dendrites were analyzed for spine density in total for both groups.

### Statistical analyses

Sample sizes were determined a priori using a power analysis with effect sizes determined from previous and/or pilot studies. Statistical analyses compared main effects of genotype and dendritic layer and therefore no formal randomization strategy was employed. However, experiments included mice from multiple litters from different breeders and included both sexes. Quantification and analyses were completed by experimenters blind to genotype. Data are presented as animal averages, unless otherwise indicated in the legend. Statistical tests are two-tailed unless otherwise indicated. A custom Python code was written to parse the segmented data from Biodock and get the aspect ratio, count, total area and distance to nearest neighbor for the dendritic mitochondria objects. Statistical analyses were done in python (v3.11) or Prism (GraphPad Prism 10) with an alpha of 0.05 considered significant. In addition to traditional statistics, a hierarchical statistical bootstrap was performed on the segmented mitochondria data as described above. Python codes for the analysis and hierarchical bootstrap of dendritic mitochondria are available in a GitHub repository: https://github.com/kpannoni/sem-mitos.

## Supplementary Information

Supplementary Material 1.

## Data Availability

Custom analysis codes and exported data from Biodock are available on GitHub: https://github.com/kpannoni/sem-mitos. Other data is available from the authors upon request. To request, contact farrissl@vtc.vt.edu.
