# Supplementary material for: MCU expression in hippocampal CA2 neurons modulates dendritic mitochondrial morphology and synaptic plasticity
Source: Sci Rep. 2025 Feb 6;15:4540. doi: 10.1038/s41598-025-85958-4 (PMC11802895; doi:10.1038/s41598-025-85958-4)
Supplement: Supplementary file 1 — Supplementary Material 1. [file 41598_2025_85958_MOESM1_ESM.pdf]

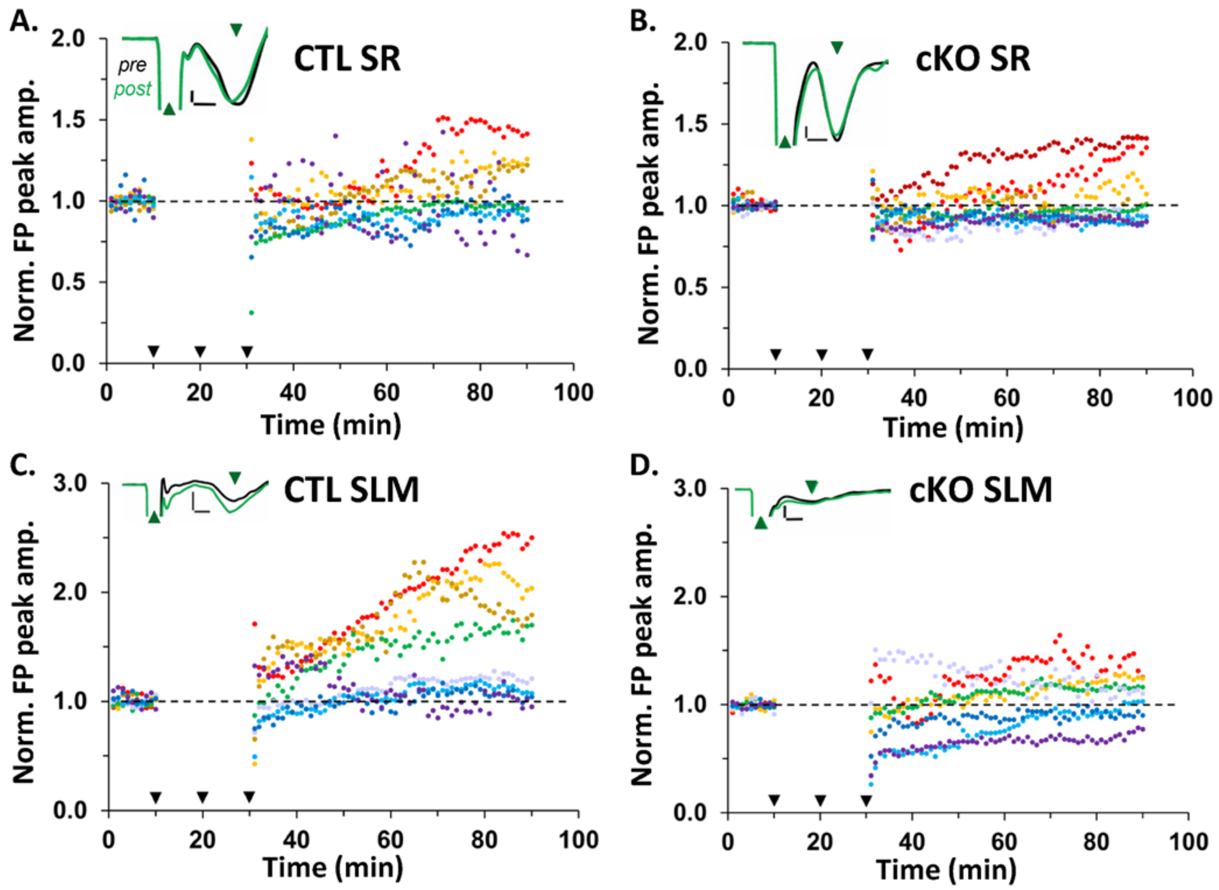

Supplemental Fig 1: Individual time plots of normalized field potential peak amplitude for each region and genotype showing the heterogeneity of responses by animal.

A. Individual time plots of normalized FP peak amplitudes evoked by stimulation of CA3 inputs to SR of CTL slices. Individual plots are color-coded according to post/pre ratio, with progressively warmer colors representing progressively higher post/pre ratios (all panels). Black dotted line = baseline (all panels). Black arrowheads = stimulation at 100 Hz for 1s; 3 bursts with 10 min interval. Inset shows a representative recording of the average evoked response recorded during the last 5 minutes of pre-conditioning (black line) versus the last 5 minutes of post-conditioning (green line; all panels). Green up arrowhead = stimulus artifact; green down arrowhead = FP. Scale bars: 0.1mV, 0.5ms (all panels).

B. Individual time plots of normalized FP peak amplitude evoked by stimulation of CA3 inputs to SR of cKO slices.

C. Individual time plots of normalized FP peak amplitude evoked by stimulation of ECII inputs to SLM of CTL slices.

D. Individual time plots of normalized FP peak amplitude evoked by stimulation of ECII inputs to SLM of cKO slices.

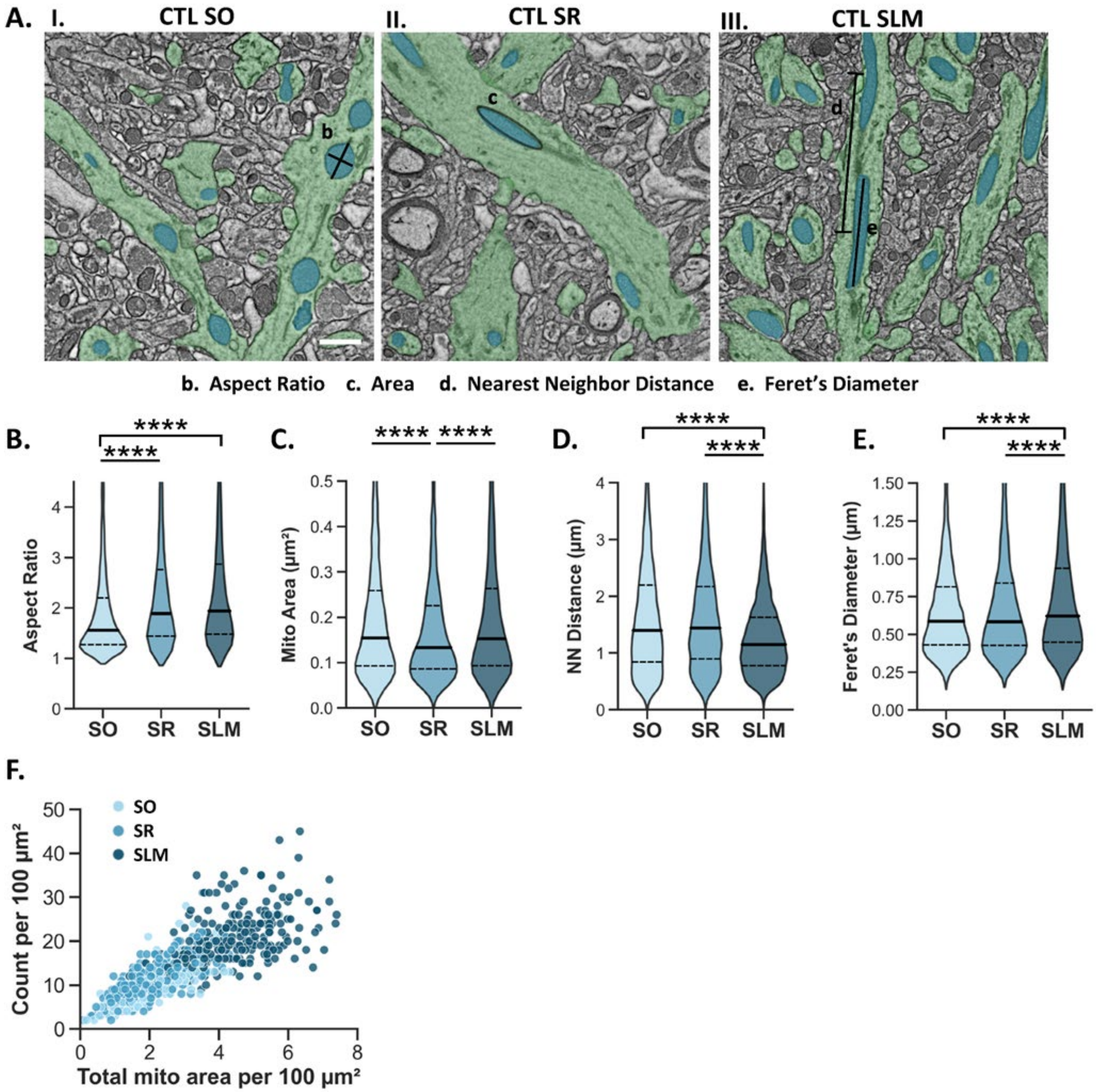

Supplemental Fig. 2: Morphometrics separate mitochondria into distinct populations within CA2 dendritic layers.

A. Representative SEM images showing segmented dendritic mitochondria (blue) and dendrites (green) in CA2 SO, SR and SLM of CTL mice. Examples of the measured metrics are illustrated. Scale bar = 1 µm for all images.

B. Mitochondrial aspect ratio in CA2 SO, SR, and SLM of CTL. SO n = 2353, SR n = 2904, SLM n = 5236 mitochondria from 3 mice). See Table 1 for summary statistics.

C. Individual mitochondria area in the same dataset as in (B).

D. Mitochondria nearest neighbor distance in the same dataset as in (B).

E. Mitochondria Feret's diameter in the same dataset as in (B).

F. A correlation of mitochondrial count and total mitochondrial area per 100 μm2 image tile in CA2 SO, SR, and SLM. SO n = 223, SR n = 279, SLM n = 260, n = 100 μm2 tiles. For all violin plots, solid line = median; dashed line = upper and lower quartiles.

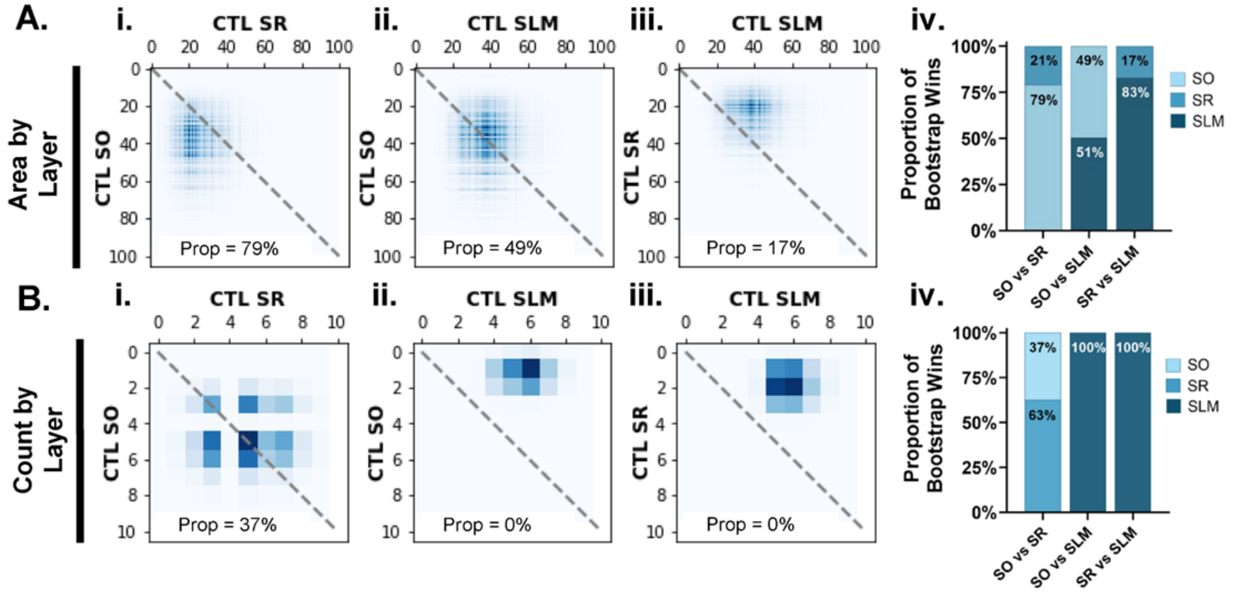

Supplemental Fig. 3: Confirmation of mitochondrial ultrastructure differences across dendritic layers of CTL CA2 with a hierarchical bootstrap.

A. Comparison of resampled mitochondria area medians across SO and SR (i), SO and SLM (ii) or SR and SLM (iii) of CTL CA2 with a probability distribution plot. Prop = the proportion of medians within the lower triangle of the plot. N = 10,000 iterations. Data were binned into 100 equal bins. (iv) Bar plot showing the proportion of bootstrap iterations where mitochondria area was greater in SO, SR or SLM for each comparison.

B. Comparison of resampled median mitochondria count per tile across SO and SR (i), SO and SLM (ii) or SR and SLM (iii) of CTL CA2. N = 10,000 iterations. Data was binned into 10 equal bins. (iv)Bar plot showing the proportion of bootstrap iterations where mitochondria count per tile was greater in SO, SR or SLM for each layer comparison in CTL CA2.

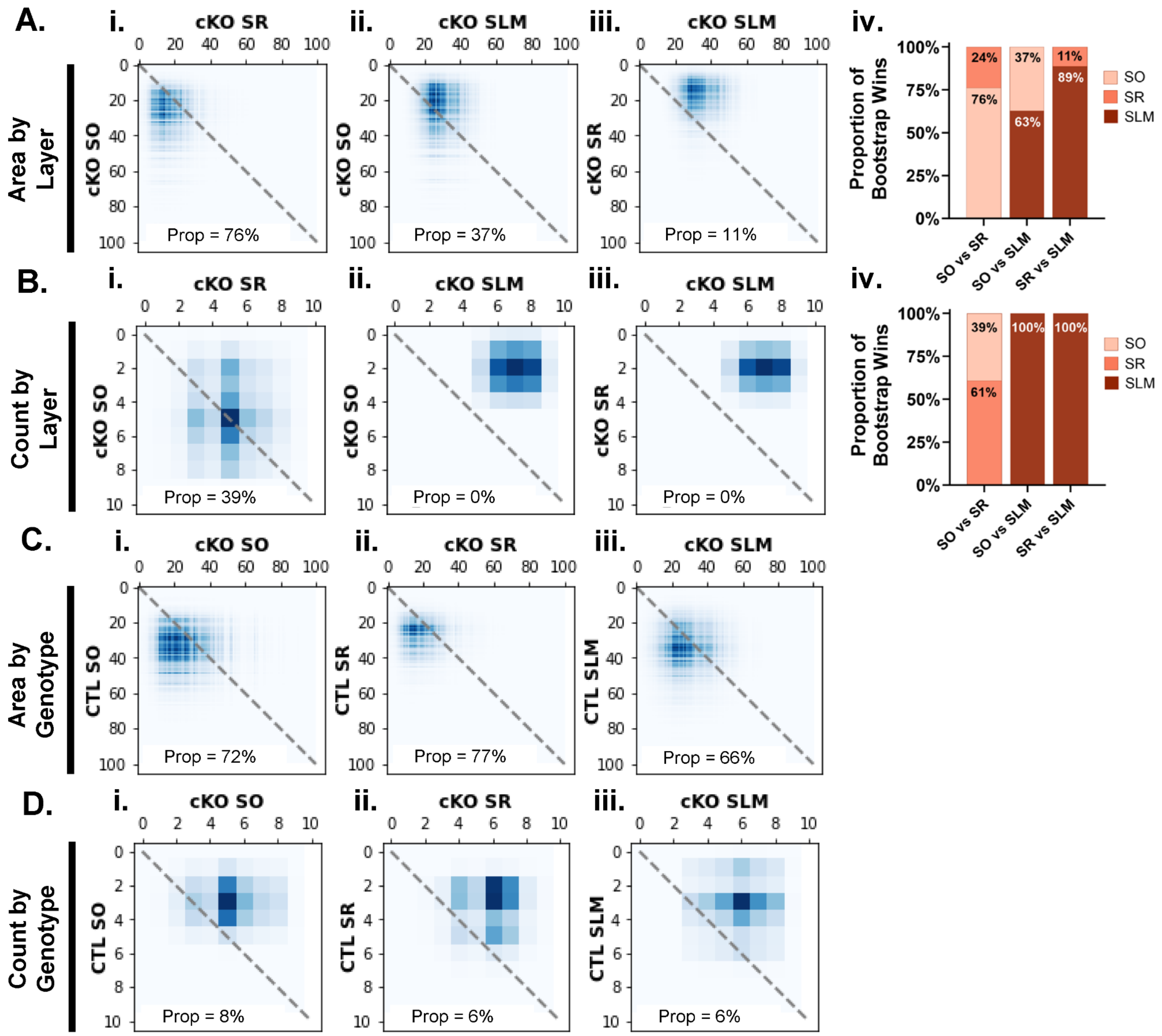

Supplemental Fig. 4: Confirmation of mitochondrial ultrastructure differences across dendritic layers of CTL and cKO mice with a hierarchical bootstrap.

A. Comparison of resampled mitochondria area medians across SO and SR (i), SO and SLM (ii) or SR and SLM (iii) of CA2 in MCU cKO mice. Prop = the proportion of medians within the lower triangle of the plot. N = 10,000 iterations. Data was binned into 100 equal bins. (iv) Bar plot showing the proportion of bootstrap iterations where mitochondria area was greater in SO, SR or SLM for each layer comparison in the cKO.

B. Comparison of resampled median mitochondria count per tile across SO and SR (i), SO and SR (ii) or SR and SLM (iii) in CA2 of MCU cKO mice. N = 10,000 iterations. Data was binned into 10 equal bins. (iv) Bar plot showing the proportion of bootstrap iterations where mitochondria count per tile was greater in SO, SR or SLM for each layer comparison.

C. Comparison of resampled mitochondria area medians in the cKO and CTL for SO (i), SR (ii) and SLM (iii). This is the same bootstrap population as in Figure 3I.

D. Comparison of resampled mitochondria count medians in the cKO and CTL for SO (i), SR (ii) and SLM (iii). This is the same bootstrap population as in Figure 3J.

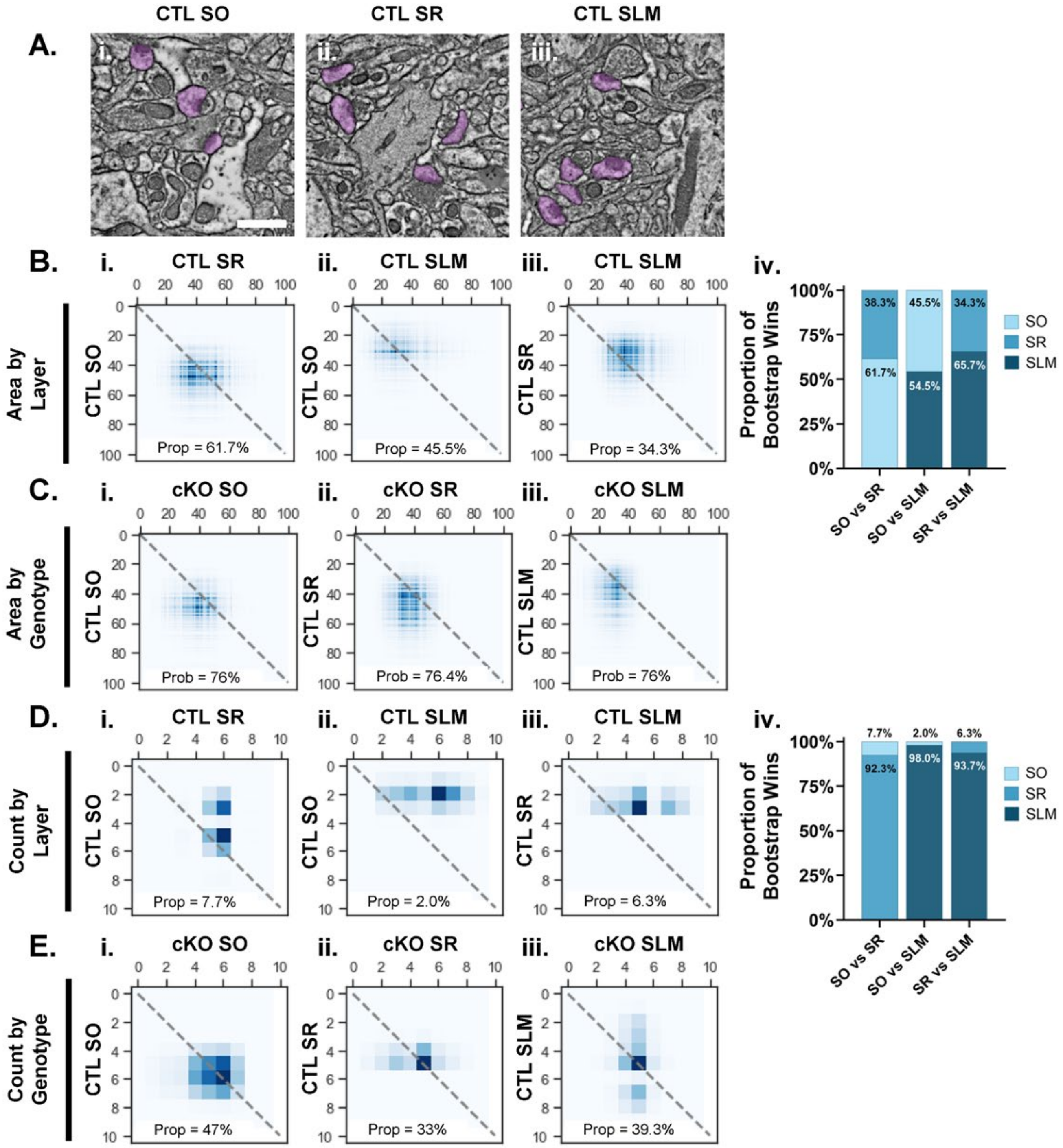

Supplemental Fig. 5: Confirmation of dendritic spine head size and count across layers and genotypes with a hierarchical bootstrap.

A. Representative SEM images from CA2 SO (i), SR (ii) and SLM (iii) of a CTL mouse. Dendritic spines are highlighted in purple. Scale = 1μm for all images.

B. Comparison of resampled median spine head area across SO and SR (i), SO and SLM (ii) or SR and SLM (iii) of CA2 in CTL mice. Prop = the proportion of medians within the lower triangle of the plot. N = 10,000 iterations. Data was binned into 100 equal bins. (iv) Bar plot showing the proportion of bootstrap iterations where spine head area was greater in SO, SR or SLM for each layer comparison in the CTL.

C. Comparison of resampled median spine head area between CTL and cKO mice in CA2 SO (i), SR (ii) and SLM (iii). Data is from the same bootstrap population as Figure 4B(ii). (iv) Bar plot showing the proportion of bootstrap iterations where spine head area was greater in CTL or cKO in SO, SR and SLM.

D. Comparison of resampled median spine head count across SO and SR (i), SO and SLM (ii) or SR and SLM (iii) of CA2 in CTL mice. Prop = the proportion of medians within the lower triangle of the plot. N = 10,000 iterations. Data was binned into 10 equal bins. (iv) Bar plot showing the proportion of bootstrap iterations where spine head count was greater in SO, SR or SLM for each layer comparison in the CTL.

E. Comparison of resampled median spine head count between CTL and cKO mice in CA2 SO (i), SR (ii) and SLM (iii). Data is from the same bootstrap population as Figure 4C(ii). (iv) Bar plot showing the proportion of bootstrap iterations where spine head count was greater in CTL or cKO in SO, SR and SLM.

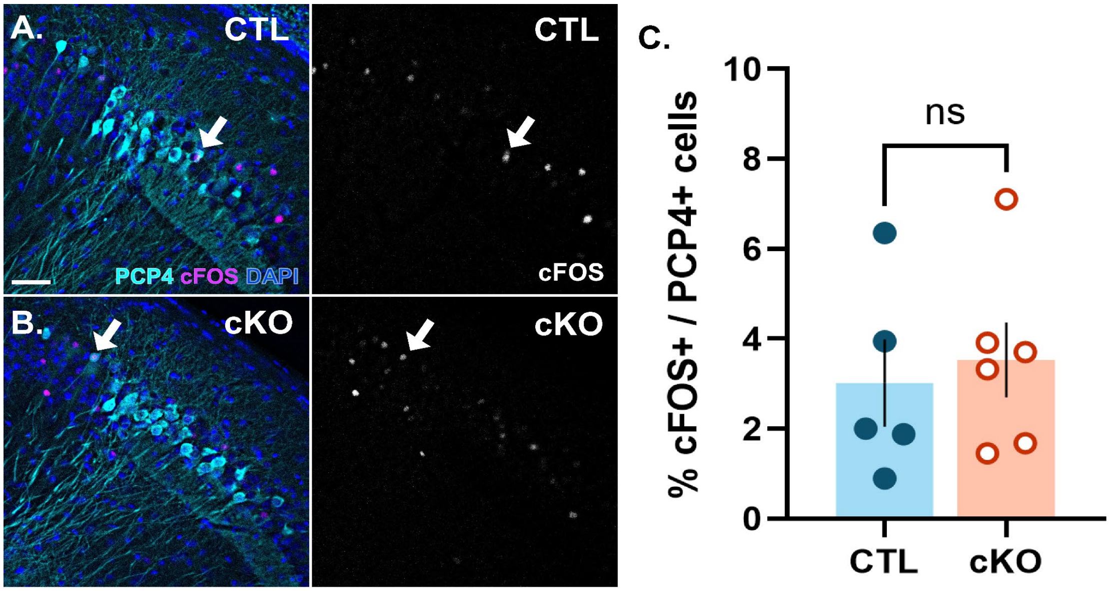

Supplemental Fig. 6: MCU cKO does not alter cFOS expression in PCP4+ CA2 neurons.

A. Representative 20X images from CA2 of a CTL mouse showing PCP4 (cyan), cFOS (magenta) and DAPI staining (blue). White arrows indicate PCP4+ / cFOS+ cells. Right: cFOS channel alone. Scale = 50μm.

B. Representative 20X images from CA2 of a cKO mouse showing PCP4 (cyan), cFOS (magenta) and DAPI staining (blue). Right: cFOS channel alone.

C. Bar plot showing the percentage of cFOS+ and PCP4+ cells over the total number of PCP4+ cells in 5 CTL (blue) and 6 cKO animals (orange). Each datapoint is the average of 4-6 sections per mouse. P = 0.70, two-tailed unpaired t-test.
